# Optimal shortening of uniform covering arrays

**DOI:** 10.1371/journal.pone.0189283

**Published:** 2017-12-21

**Authors:** Jose Torres-Jimenez, Nelson Rangel-Valdez, Himer Avila-George, Oscar Carrizalez-Turrubiates

**Affiliations:** 1 Information Technology Laboratory, CINVESTAV-Tamaulipas. Cd. Victoria, Tamaulipas 87130, México; 2 Instituto Tecnológico de Ciudad Madero, CONACYT-TecNM. Cd. Madero, Tamaulipas 89440, México; 3 Unidad de Transferencia Tecnológica Tepic, CONACYT-CICESE, Tepic, Nayarit 63173, México; 4 SVAM International de México. Cd. Victoria, Tamaulipas 87130, México; Bangladesh University of Engineering and Technology, BANGLADESH

## Abstract

Software test suites based on the concept of interaction testing are very useful for testing software components in an economical way. Test suites of this kind may be created using mathematical objects called covering arrays. A covering array, denoted by *CA*(*N*; *t*, *k*, *v*), is an *N* × *k* array over $Z_{v}=\left\{0,\ldots ,v-1\right\}$ with the property that every *N* × *t* sub-array covers all *t*-tuples of $Z_{v}^{t}$ at least once. Covering arrays can be used to test systems in which failures occur as a result of interactions among components or subsystems. They are often used in areas such as hardware Trojan detection, software testing, and network design. Because system testing is expensive, it is critical to reduce the amount of testing required. This paper addresses the Optimal Shortening of Covering ARrays (OSCAR) problem, an optimization problem whose objective is to construct, from an existing covering array matrix of uniform level, an array with dimensions of (*N* − *δ*) × (*k* − Δ) such that the number of missing *t*-tuples is minimized. Two applications of the OSCAR problem are (a) to produce smaller covering arrays from larger ones and (b) to obtain quasi-covering arrays (covering arrays in which the number of missing *t*-tuples is small) to be used as input to a meta-heuristic algorithm that produces covering arrays. In addition, it is proven that the OSCAR problem is NP-complete, and twelve different algorithms are proposed to solve it. An experiment was performed on 62 problem instances, and the results demonstrate the effectiveness of solving the OSCAR problem to facilitate the construction of new covering arrays.

## Introduction

Functionality tests during software development demand special attention, and they are generally important for preventing malfunctions in software components. During the testing phase, it is desirable to find all errors that could arise in a software component before it is delivered to the user. If a software component has a large number of parameters, then testing it exhaustively might be expensive because of the large number of configurations that can arise from the different parameters’ values; e.g., a software component with just 20 parameters of 2 different values each would require 2^20^ = 1,048,576 tests. An alternative is to test the system using a small, randomly generated test suite, but in this case, there is no guarantee of the testing coverage; instead, a better choice is to use a combinatorial testing approach that provides a coverage guarantee for small test suites. This combinatorial testing approach (also called interaction testing) guarantees the coverage of all interactions of a certain size among different values of the input parameters of a software component. This approach is based on evidence presented by [1] that many errors are produced by the interactions of only a few parameter values. Specifically, the cited authors showed evidence that test suites with an interaction size of 6 are sufficient to detect all known errors in a collection of different software components.

A uniform covering array (CA), denoted by *CA*(*N*;*t*, *k*, *v*), is a commonly used structure in interaction testing. It is an array C with dimensions of *N* × *k* constructed over $Z_{v}=\left\{0,\ldots ,v-1\right\}$ with the property that every *N* × *t* sub-array covers all members of $Z_{v}^{t}$ at least once. The value of *N* is the number of rows of C, i.e., the number of test cases; *k* is the number of columns or parameters; *v* is the number of values that each parameter can take; and *t* is the degree of interaction among the parameters. Because there are (kt) sets of *t* columns {*c*_1_,…,*c*_*t*_}, the number of different *t*-tuples that must be covered at least once in C is vt(kt). When a specific *t*-tuple is missing in a set of *t* columns (*c*_1_, …, *c*_*t*_), we refer to it as a missing *t*-wise combination (or a missing combination, for short). Below, a *CA*(6; 2, 5, 2) in which all 22(52) *t*-wise combinations are covered at least once is shown.

$$\begin{array}{c} C=(\begin{array}{ccccc} 0 & 0 & 0 & 0 & 0\\ 1 & 1 & 1 & 1 & 1\\ 1 & 1 & 1 & 0 & 0\\ 1 & 0 & 0 & 1 & 1\\ 0 & 1 & 0 & 1 & 0\\ 0 & 0 & 1 & 0 & 1 \end{array}) \end{array}$$

The covering array construction (CAC) problem is the search for the covering array number (CAN), i.e., the minimum value *N* for which an array *CA*(*N*;*t*, *k*, *v*) still exists. Formally, the CAN can be defined as *CAN*(*t*, *k*, *v*) = min{*N*|∃ *CA*(*N*; *t*, *k*, *v*)}.

In some theoretical studies, the following definition is adopted: *CAN*(*t*, *k*, *v*) = *O*(*v*^*t*^ log *k*) [2]. This definition is interesting because as the number of columns grows linearly, the number of rows grows only logarithmically. This is an advantage of such combinatorial structures because of the possibility of deriving small test suites. For instance, for a software component with 126 binary parameters, exhaustive testing would require 2^126^ tests, whereas interaction testing with strength 2 would require only 10 tests.

A complementary problem to the CAC problem is known as the test suite reduction problem (TSRP), which consists of finding, for a given array, the smallest subset of rows that covers all *t*-wise combinations [3]. The CAC problem is a special case of the TSRP in which the input is an array that contains all *v*^*k*^ distinct test cases.

For some special cases, there are algorithms that can solve the CAC problem in polynomial time:

- when *v* = *t* = 2 [4],
- when *v* is a prime power and *k* ≤ *v* + 1 [5], and
- when *k* = *t* + 1 [6].

However, the CAC problem remains highly combinatorial in most cases. Moreover, some variants have been proven to be NP-complete; e.g., the work presented in [2, 7] shows the NP-completeness of the problem of extending a matrix by one row with no fewer than *m* missing *t*-wise combinations. The problem defined in the current work is also NP-complete, as proven in this paper.

Various methods have been developed to address the CAC problem. Exact methods solve it to optimality; however, they usually require exponential time to achieve their goal [8–11]. As a result of this complexity, various approximate methods have been proposed as alternatives, including recursive [4, 12, 13], algebraic [14, 15], greedy [16–20], and meta-heuristic approaches. This last category includes methods based on strategies such as genetic algorithms [21], simulated annealing [22], and tabu search [23].

These approximate algorithms can be used to build non-optimal CAs in a reasonable time; some of these algorithms depend on the quality of their inputs to produce small CAs. Most of the time, these inputs are based on matrices that are nearly CAs. The objective of the present work is to construct matrices with sufficiently few missing combinations to still be considered quasi-CAs. Such arrays are created by solving the problem known as the Optimal Shortening of Covering ARrays (OSCAR); related results were published in [24]. The OSCAR problem is relevant to the construction of CAs because it can produce smaller CAs or excellent initialization matrices for meta-heuristic algorithms for constructing CAs. The main contributions of this work are as follows. It formalizes three of the five algorithms presented in [24]. It also presents seven new approximate strategies for solving the OSCAR problem. In addition, the present work offers a complete analysis of the performance of all of the new and old algorithms, something that has not been done before. Furthermore, it proposes three new benchmarks with more than 800 OSCAR instances, which extend the range of study to matrices with strengths of *t* = {2, 3, 4, 5}, whereas previous works have studied only *t* = 2; these benchmarks are used as part of the experiments conducted to analyze the strategies. These experiments not only evaluate how effectively the algorithms solve the OSCAR problem but also compare the best of them against state-of-the-art strategies. These experiments provide evidence that solving the OSCAR problem using the proposed approaches enables the creation of quasi-CAs that are better than other reported initialization functions and even than the fast and versatile IPOG-F, a state-of-the-art algorithm for constructing CAs; the main result is that the arrays produced using the proposed algorithms have 90% fewer missing *t*-wise combinations than those generated using the other approaches considered for comparison.

This paper is organized as follows. In the problem definition section, the OSCAR problem is formally defined; its NP-completeness is proven, and some of its applications are described. In the related work section, some of the work related to initialization functions for meta-heuristics for CA construction is presented. Subsequently, the algorithms proposed in this work for solving the OSCAR problem are presented. In the experimentation section, an experiment performed to test the proposed algorithms for the construction of matrices with few missing combinations is presented. Finally, in the conclusions section, final comments regarding this work are provided.

## Problem definition

Let A denote a *CA*(*N*; *t*, *k*, *v*) or a quasi-*CA*(*N*; *t*, *k*, *v*) (a quasi-CA is a matrix with a relatively small number of missing *t*-combinations). Then, the OSCAR problem can be defined as $\text{min}\{\tau \left(B_{N^{\prime }\times k^{\prime }}\right)|B\;\text{is}\;\text{a}\;\text{submatrix}\;\text{of}\;A\}$, where τ(B) is a function that counts the number of missing *t*-wise combinations in the given array and *N*′ = *N* − *δ* and *k*′ = *k* − Δ are defined in terms of two predefined integer values, 0 ≤ *δ* ≤ *N* − *v*^*t*^ and 0 ≤ Δ ≤ *k* − *t*, which satisfy *δ* > 0 ∨ Δ > 0. Hence, an OSCAR instance is specified by the elements (A,δ,Δ).

The search space for an OSCAR instance consists of all submatrices B of the given matrix A. Accordingly, the number of feasible solutions that form such a space can be estimated to be (NN-δ)(kk-Δ), where (NN-δ) and (kk-Δ) represent the numbers of different ways to choose subsets of rows and columns, respectively, from the original matrix A. Throughout the remainder of this document, for a given submatrix B, we use $J_{R}$ to denote the subset of rows chosen from A and $J_{C}$ to denote the subset of columns.

We present an example of a solution to the OSCAR instance specified by A=CA(6;2,5,2) (see Table 1), *δ* = 2, and Δ = 2, for which it is feasible to construct a solution B (see Table 2) where τ(B)=0. The solution for this instance is obtained by eliminating $J_{R}=\left\{0,2,4,5\right\}$ and $J_{C}=\left\{2,3,4\right\}$ from A. Because τ(B)=0, the solution B is a *CA*(4; 2, 3, 2).

**Table 1:** OSCAR example, the input array A=CA(6;2,5,2).

$\left(\begin{array}{ccccc} 0 & 0 & 0 & 0 & 0\\ 1 & 1 & 1 & 0 & 0\\ 1 & 0 & 1 & 0 & 1\\ 0 & 0 & 0 & 1 & 1\\ 1 & 1 & 0 & 1 & 1\\ 0 & 1 & 1 & 1 & 0 \end{array}\right)$

**Table 2:** Solution to the OSCAR problem, B=CA(4;2,3,2), when *δ* = 2 and Δ = 2.

$\left(\begin{array}{ccc} 0 & 0 & 0\\ 1 & 0 & 1\\ 0 & 1 & 1\\ 1 & 1 & 0 \end{array}\right)$

Alternatively, the matrix A can be represented by another matrix $A^{\prime }$ with dimensions of N×(kt). This matrix has the same number of rows as A and contains one column for each subset of *t* columns derived from A. Each cell $a_{i,j}^{\prime }\in A^{\prime }$ contains a value from the set {0, 1, …, *v*^*t*^ − 1}; this value represents the *t*-tuple covered by row *i* in the subset of *t* columns associated with column *j*.

The OSCAR instance (A,δ,Δ)=(CA(4;2,3,2),1,0) is shown in Tables 3, 4 and 5. The initial matrix A is shown in Table 3, the *t*-tuples and sets of columns are shown in Table 4, and the new matrix representation $A^{\prime }$ is presented in Table 5 (t-wise combinations covered).

**Table 3:** An instance of the OSCAR problem, initial matrix.

	*c*_1_	*c*_2_	*c*_3_
*r*_1_	0	0	0
*r*_2_	0	1	1
*r*_3_	1	0	1
*r*_4_	1	1	0
*r*_5_	1	1	1

**Table 4:** An instance of the OSCAR problem, the *t*-tuples and sets of columns.

*t*-tuples	set of *t* columns
0. (0,0)	*t*_1_ = (*c*_1_, *c*_2_)
1. (0,1)	*t*_2_ = (*c*_1_, *c*_3_)
2. (1,0)	*t*_3_ = (*c*_2_, *c*_3_)
3. (1,1)	

**Table 5:** An OSCAR instance, t-wise combinations covered.

	*t*_1_	*t*_2_	*t*_3_
*r*_1_	0	0	0
*r*_2_	1	1	3
*r*_3_	2	3	1
*r*_4_	3	2	2
*r*_5_	3	3	3

Finally, the tuple $\left(A^{\prime },\delta ,\Delta \right)$ is used to define an instance of the OSCAR problem, and the NP-completeness of the problem can be proven based on this new representation. The remainder of this section is devoted to this proof.

### The proof that the OSCAR problem is NP-complete

To demonstrate the NP-completeness of the OSCAR problem, it is necessary to show it to be equivalent to a problem that is already known to be NP-complete. For this purpose, this work presents the transformation of the maximum cover (or MAXCOVER) problem (cf. [25] for a review of this problem) into the OSCAR problem. For the proof, the previously defined notation $\left(A^{\prime },\delta ,\Delta \right)$ for an OSCAR instance is extended to $\left(A^{\prime },\delta ,\Delta ,h\right)$, where the value *h* denotes an integer that supports the following question: is there a sub-array B of $A^{\prime }$ with dimensions of (*N* − *δ*) × (*k* − Δ) such that τ(B)≤h? This question transforms the OSCAR problem into its decision form, which is required for this demonstration.

First, it is proven that the OSCAR problem is NP in nature. Let us begin with the case in which Δ = 0, meaning that the matrix B is a subset of only the rows of $A^{\prime }$. Clearly, the size of the search space is reduced to (NN-δ). The claim that the problem is NP in nature holds because computing the value of τ(B) would require time proportional to O(N×(kt)) to examine all possible *t*-wise combinations, which are equal in number to the number of columns of B. In other words, the question of whether τ(B)≤h for the OSCAR problem can be answered in polynomial time in the dimensions of B.

Now that it has been shown that the OSCAR problem is NP in nature, let us proceed with the transformation of the NP-complete MAXCOVER problem. The objective of the MAXCOVER problem is to cover a given set $Q=\{q_{1},q_{2},\ldots ,q_{l}\}$, regarded as the universe. To achieve this goal, we must use a subset of $Y=\{Y_{1},Y_{2},\ldots ,Y_{m}\}$, where each subset $Y_{i}\subseteq Q$, for all 1 ≤ *i* ≤ *m*, is given in advance and has a size of at most *C*. This problem can be characterized by the tuple (Q,Y,C) and can be transformed into an OSCAR instance $\left(A^{\prime },\delta ,\Delta ,l\right)$ as follows: a) The matrix $A^{\prime }$ is constructed, with *m* + 1 rows and *l* + max{|*Y*_*i*_|} + 1 columns. b) For 1 ≤ *i* ≤ *m* and 1 ≤ *j* ≤ *l*, the value $a_{i,j}^{\prime }$ of each cell is 1 if subset *Y*_*i*_ covers element *q*_*j*_ or 0 otherwise. c) For 1 ≤ *i* ≤ *m* and *j* > *l*, the value $a_{i,j}^{\prime }$ of each cell is 0. d) For *i* = *m* + 1, the value $a_{i,j}^{\prime }$ of each cell is 0 if 1 ≤ *j* ≤ *l* or 1 otherwise. e) The values of *δ*, Δ, and *h* are set to *m* − *C*, 0, and 0, respectively. The matrix $A^{\prime }$ can be constructed in a time of *O*(*lm*), and the derived OSCAR instance is denoted by $\left(A^{\prime },\delta ,0,0\right)$.

Table 6 shows an example of the transformation of the MAXCOVER problem into the OSCAR^1^ problem. The following elements are used in this case:

- $Q=\{q_{1},q_{2},q_{3},q_{4},q_{5}\}$
- $Y=\{Y_{1}=\left\{q_{1},q_{2},q_{5}\right\}$
- *Y*_2_ = {*q*_2_, *q*_4_, *q*_5_}, *Y*_3_ = {*q*_1_, *q*_4_, *q*_5_}, *Y*_4_ = {*q*_1_, *q*_2_, *q*_3_}, *Y*_5_ = {*q*_2_, *q*_3_, *q*_4_}
- *C* = 3

**Table 6:** The MAXCOVER instance specified by $Q=\{q_{1},q_{2},q_{3},q_{4},q_{5}\}$, $Y=\{Y_{1}=\left\{q_{1},q_{2},q_{5}\right\}$, *Y*_2_ = {*q*_2_, *q*_4_, *q*_5_}, *Y*_3_ = {*q*_1_, *q*_4_, *q*_5_}, *Y*_4_ = {*q*_1_, *q*_2_, *q*_3_}, *Y*_5_ = {*q*_2_, *q*_3_, *q*_4_}}, and *C* = 3 represented as an OSCAR instance.

Row	$A^{\prime }$								
	*q*_1_	*q*_2_	*q*_3_	*q*_4_	*q*_5_	*q*_6_	*q*_7_	*q*_8_	*q*_9_
*Y*_1_	1	1	0	0	1	0	0	0	0
*Y*_2_	0	1	0	1	1	0	0	0	0
*Y*_3_	1	0	0	1	1	0	0	0	0
*Y*_4_	1	1	1	0	0	0	0	0	0
*Y*_5_	0	1	1	1	0	0	0	0	0
*Y*_6_	0	0	0	0	0	1	1	1	1

The array $A^{\prime }$ has dimensions of 6 × 9, and the *δ* is equal to 5 − 3 = 2.

Finally, to complete the proof that the OSCAR problem is NP-complete, we demonstrate that the OSCAR instance $\left(A^{\prime },\delta ,0,0\right)$ built from the MAXCOVER instance (Q,Y,C) has a solution if and only if the latter has a solution. For this purpose, we start by showing that an optimal solution for $\left(A^{\prime },\delta ,0,0\right)$ must include row *Y*_6_ of $A^{\prime }$. This fact can be easily proven since all *t*-tuples must be covered in $\left(A^{\prime },\delta ,0,0\right)$ and those with the value 1 in any column *j* > *l* can only be covered by row *Y*_*m*+1_.

The next step is to show that there is a solution with *C* subsets for the MAXCOVER instance iff there is a matrix with *C* + 1 rows that solves $\left(A^{\prime },\delta ,0,0\right)$. This condition can also be easily proven. We first note that the *t*-tuple with value 0 is covered for any column *j* ≤ *l* by the row *Y*_*m*+1_. The same tuple is also covered for any column *j* > *l* by any row from {*Y*_1_,…,*Y*_*m*_}. With this information, the only *t*-tuples that remain uncovered are those with value 1 in any column *j* ≤ *l*. Given that during the construction of the OSCAR instance, a *t*-tuple with value 1 is assigned only to those rows in columns *j* ≤ *l* that are associated with a subset of Y, the following claim is valid: any subset of Y that is formed of *C* elements and represents a solution for the MAXCOVER instance can also be transformed into a solution for the OSCAR instance. This claim is justified since the associated rows with the chosen *C* elements cover all *t*-tuples for any column but those with value 0 in columns *j* > *l*. Then, it is necessary only to add row *m* + 1 to cover the missing *t*-tuples. It is also true that a solution with *C* + 1 rows for the OSCAR instance is a valid solution for the equivalent MAXCOVER instance, since it is necessary only to choose those subsets of Y associated with the rows selected in the solution for the OSCAR instance. Finally, if one of these instances has no solution, then neither does the other; this claim holds because of the equivalence between such solutions, which has already been shown. Hence, it is demonstrated that a solution to the MAXCOVER problem implies a solution to the OSCAR problem.

Finally, any instance of the OSCAR problem for the case of Δ > 0 is equivalent to (kk-Δ) instances of the problem with Δ = 0. Since it has been proven that instances of this special case are NP-complete, then the general case of the OSCAR problem is at least as complex.

### Applications of the OSCAR problem

Methods of solving the OSCAR problem have the following applications: a) they can reduce the search space in the CAC problem; b) they can directly construct CAs, when there are no *t*-wise combinations missing in the matrices they generate; c) they can be used as initializing functions for meta-heuristics for CA construction; d) they can aid in the identification of better upper bounds for CA matrices; and e) they can be used for fine-tuning in experimental design. Each of these applications is detailed in the remainder of this section.

The OSCAR problem successfully yields a quasi-CA that has zero a small number of missing *t*-wise combinations. Such a situation is convenient since instead of searching for a *CA*(*N* + *δ*; *t*, *k* + Δ, *v*) in a feasible region with a size of O((vkN)), corresponding to the original domain, it may be possible to construct such a CA from a relaxed region of a smaller size, (N+δδ)(k+ΔΔ).

The second and third applications of the OSCAR problem are related to the construction of CAs. The OSCAR problem enables the direct construction of CAs when τ(B)=0, i.e., when B is a CA. Additionally, whenever the matrix constructed as a solution to an OSCAR instance is not a CA (i.e., the number of missing *t*-tuples is greater than zero), this solution can still be used indirectly for CA construction because it can serve as the initial solution for meta-heuristic algorithms. Note that the performance of a meta-heuristic for constructing CAs depends on the quality of the initial matrix. Hence, the sub-array obtained as a solution to the OSCAR problem is adequate for this purpose because it has only a few missing *t*-wise combinations; this is in contrast to arrays of the same size constructed using random initialization functions, which are likely to be missing a large number of the possible *t*-wise combinations due to their random nature. Some of the existing meta-heuristic algorithms designed for CA construction, which show dependence on the initial matrix, are reported in [21, 23, 26, 27]. It is in algorithms of this type that the OSCAR problem finds its main area of application, namely, the generation of initial matrices with few missing *t*-wise combinations.

The fourth application of the OSCAR problem is the identification of new upper bounds for CA matrices. Many such upper bounds have been reported in the literature. For example, the best upper bounds for some CAs can be found in the repositories of [28, 29]. In addition, some bounds on *CAN*(*t*, *k*, *v*) can be found in [29]; however, the corresponding CAs have values of *N* that are far from optimal.

Because of the hardness of the CAC problem, the value of *CAN*(*t*, *k*, *v*) for any arbitrary set of values of *t*, *k*, and *v* is generally unknown. However, suitable new upper bounds can be obtained from existing matrices; e.g., between *CA*(174; 2, 110, 9) and *CA*(177; 2, 117, 9), the upper bounds on the required numbers of columns for the cases of *N* = 175 and *N* = 176 are unknown, but it can be inferred that they should be between 111, …, 116. Because most of these upper bounds have not been shown to be optimal, the question arises as to whether other upper bounds can be found. We conclude that inputs derived by solving the OSCAR problem can be used to test potential upper bounds in order to find new bounds for *CA*(*N*; *t*, *k*, *v*); this can be achieved through the proper selection of the values *δ* and Δ used to reduce the matrix size.

Some specific cases of the values of *δ* and Δ are as follows:

1. When *δ* > 0 and Δ = 0, i.e., only the number of rows is to be reduced, the rows that are selected to be discarded are those whose elimination results in the minimum number of missing combinations in the final array.
2. When *δ* = 0 and Δ > 0, i.e., only the columns of columns is to be reduced, the columns that are selected to be discarded are similarly those whose elimination results in the minimum number of missing *t*-wise combinations. However, this case makes sense only when the array A is not a CA.

Finally, another application of solutions to the OSCAR problem is their direct use in testing scenarios. Through the careful selection of the OSCAR problem parameters *δ* and Δ, it is possible to ensure that resulting sub-array has the desired numbers of rows (i.e., test cases) and columns (i.e., parameters) to produce a quasi-CA (with 90–100% coverage of the *t*-tuples) that provides the required level of assurance.

The proposed methodology for the construction of CAs consists of generating an initial solution for a meta-heuristic algorithm by solving an instance of the OSCAR problem; i.e., the OSCAR problem is solved to obtain the solution B, which is then used as the initial array in a meta-heuristic algorithm.

## Related work

The construction of CAs is a highly combinatorial problem that can benefit from the use of approximate algorithms to construct CAs of a desired size within a reasonable amount of time. Many researchers, instead of directing their efforts toward finding CAs with the minimum number of rows using an exact approach, have designed approximate algorithms to improve the best known *upper bound* for CAs and then reduce the gap between that bound and the CAN. These CA construction algorithms can be classified, in accordance with their characteristics, into the following types: (a) algebraic approaches, (b) exact approaches, (c) greedy approaches, (d) transformations, and (e) meta-heuristic approaches.

Algebraic methods have the characteristic that the CA construction process involves formulas or operations using mathematical objects such as vectors, finite fields, groups, and CAs with small values of *t*, *k*, and *v*. Some algebraic methods yield optimal constructions, including the *CA*(*N*; 2, *k*, 2) methods of [30] and [31]; Bush’s construction method for *CA*(*N*; *t*, *q* + 1, *q*), where *q* is a prime or a prime power and *q* ≤ *t* (cf. [5]); and the zero-sum method of [6], which yields an optimal *CA*(*t*, *t* + 1, *v*) for any *t* ≥ 2. The main feature of these approaches is that most of them require small CAs or quasi-CAs from which to construct larger CAs.

Exact methods are exhaustive approaches for the construction of optimal CAs. Although some approaches include techniques for accelerating the search process, they generally require exponential time to complete their task, making them practical only for the construction of small optimal CAs. This category includes branch-and-bound (B&B) strategies, such as the work proposed by [10], which incorporates symmetry-breaking techniques, partial *t*-wise verification and fixed blocks in the bounding process, and the work of [8], which, for the generation of a non-isomorphic *CA*(*N*; 2, *k*, 2), uses a pruning strategy based on bounds defined by the minimum ranks established in terms of the CA size.

Greedy strategies are commonly used for combinations of the parameters *N*, *t*, *k*, and *v* for which exact methods are impractical, with the basic purpose of producing a good solution in a short time. The majority of commercial and open-source tools for generating test data (including AETG [32], TCG [17], ACTS [33], IPOG-F [19], and DDA [34]) use greedy algorithms for CA construction.

Transformations generally exploit the structure of existing CAs either to make them smaller or to support other approaches, e.g., algebraic approaches, in creating smaller CAs. This task is usually performed in one of two ways: a) through the identification of redundancy or b) through the construction of submatrices. Redundancy in a CA can be identified through the permutation of rows or columns or through the changing of symbols (cf. [35], [36] and [37]). However, approaches based on the construction of submatrices provide a better basis for new CAs, and the present work can be considered to be of this type.

Finally, similar to greedy methods, meta-heuristic approaches are strategies that are not guaranteed to find a CA with the minimum number of rows. In practice, meta-heuristic methods yield very good results, but they consume more CPU time than greedy algorithms. Some meta-heuristics that have been used to solve the CAC problem include simulated annealing (SA) [22], tabu search (TS) [38], memetic algorithms (MAs) [27], and genetic algorithms (GAs) [21].

For all of the strategies described above, the main goal is the construction of CAs, i.e., matrices with zero missing *t*-wise combinations. However, the CA construction performance of algebraic and meta-heuristic approaches is improved when the initial matrices are quasi-CAs, i.e., when they are missing only a small number of the possible *t*-wise combinations. This situation raises the question of how an initial matrix should be constructed for these approaches. The answer is to use initialization functions. Hence, these initialization functions are a key element of the development of meta-heuristics for CA construction.

The main initialization functions used in state-of-the-art methods are as follows: a) random matrix initialization [21, 23, 26, 27], b) initialization with a balanced number of symbols per column [27], c) initialization through row augmentation [39], d) initialization based on submatrices [40], and e) initialization based on greedy strategies [41, 42]. The four first strategies do not consider the number of missing *t*-wise combinations in the construction of the initial matrix. Strategies of the last type can be used to build CAs, but they are typically larger than the required matrix size; this situation results in random discarding of rows and/or columns that is also not optimized in terms of the number of missing *t*-wise combinations. Hence, an alternative is to use an existing matrix of greater size and optimize the row/column reduction process until a matrix of the required size is obtained. This optimization is exactly equivalent to solving the OSCAR problem, and this work proposes a wide variety of new meta-heuristic and hybrid strategies for this purpose.

In summary, whereas CA construction approaches (e.g., exact, greedy, algebraic, meta-heuristic and transformation methods) produce matrices with no missing *t*-wise combinations, the strategies presented in this work solve the OSCAR problem to generate quasi-CAs. Quasi-CAs are important because they can be used as initial matrices for CA construction strategies based on algebraic and meta-heuristic methods and can thus improve the performance of these methods in the construction of new CAs.

**Fig 1:**
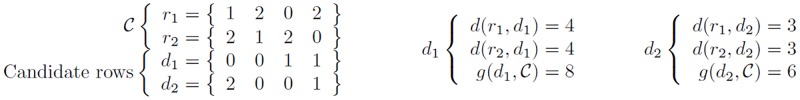
Example of the Hamming distances between the two rows *r*_1_ and *r*_2_ that are already in the matrix *C* and the two candidate rows *d*_1_ and *d*_2_.

The remainder of this section provides a more detailed introduction to some of the relevant initialization functions found in the scientific literature related to this topic. These four initialization functions will be denoted by $I_{1}$, $I_{2}$, $I_{3}$, and $I_{4}$ in this paper; Fig 1 shows an example of each one initialization function.

Each of the four initialization functions creates an array C with *N* rows and *k* columns, in which each cell is initialized with a symbol of the given alphabet {0, 1, …, *v* − 1} of *v* symbols. The function $I_{1}$ is presented in [21, 23, 26, 27]; this function initializes each cell *c*_*ij*_ of $C_{N\times k}$ with a symbol drawn at random from the set {0, 1, …,*v* − 1}. Fig 1 (random) shows an example of the use of $I_{1}$ to initialize a matrix $C_{10\times 4}$.

The function $I_{2}$ initializes $C_{N\times k}$ with a balanced number of randomly generated symbols per column. Each column *k*_*i*_, where 1 ≤ *i* ≤ *k*, will contain an almost uniform distribution of the symbols {0, 1, …, *v* − 1}. To achieve such uniformity, a symbol is generated at random for each of the *N* rows of column *k*_*i*_, but during the random generation process, it is ensured that the first $R_{1}=v-\left(N-\left\lfloor \frac{N}{v}\right\rfloor v\right)$ symbols appear $\left\lfloor \frac{N}{v}\right\rfloor$ times and that the remaining $R_{2}=N-\left\lfloor \frac{N}{v}\right\rfloor v$ symbols appear $\left\lceil \frac{N}{v}\right\rceil$ times. For example, in a 10 × 4 matrix C with an alphabet size of *v* = 3, each of the four columns contains $R_{1}=3-\left(10-\left\lfloor \frac{10}{3}\right\rfloor 3\right)=2$ symbols that appear $\lfloor \frac{10}{3}\rfloor =3$ times and $R_{2}=10-\left\lfloor \frac{10}{3}\right\rfloor 3=1$ symbol that appears $\lceil \frac{10}{3}\rceil =4$ times; this situation is exemplified in Fig 1 (balanced). The use of $I_{2}$ guarantees that each column has a balance in the cardinalities of each symbol, something that cannot be guaranteed when using $I_{1}$. The function $I_{2}$ is a generalization of the initialization function presented in [27] for solving the binary CAC problem using an SA approach.

The function $I_{3}$ initializes $C_{N\times k}$ one row at a time. This function generates the first row *r*_1_ at random; i.e., each of its cells will contain a symbol randomly chosen from {0, 1, …,*v* − 1}. Subsequently, each new row is selected from a set of two random candidate rows *d*_1_ and *d*_2_ and is added to C. The chosen candidate row is the one that maximizes the Hamming distance with respect to all rows *r*_*s*_ that already exist in C. The Hamming distance between two rows is equal to the number of positions at which the corresponding symbols are different; correspondingly, the Hamming distance between a candidate row *d*_*j*_ and all rows already in C is equal to the number of positions *l* in each row *r*_*s*_ that differ from the corresponding positions in *d*_*j*_, summed over all existing rows *r*_*s*_. Formally, this latter definition can be expressed as $g\left(d_{j},C\right)=\sum_{s=0}^{i-1}\sum_{l=0}^{k-1}h\left(r_{s,l},d_{j,l}\right)$, where *i* is the number of rows already added to C and *h*(*r*_*s*,*l*_, *d*_*j*,*l*_) = 1 if *r*_*s*,*l*_ ≠ *d*_*j*,*l*_ or 0 otherwise. This process is repeated until all *N* rows have been created. This initialization function has been used previously in [39].

**Fig 2:**
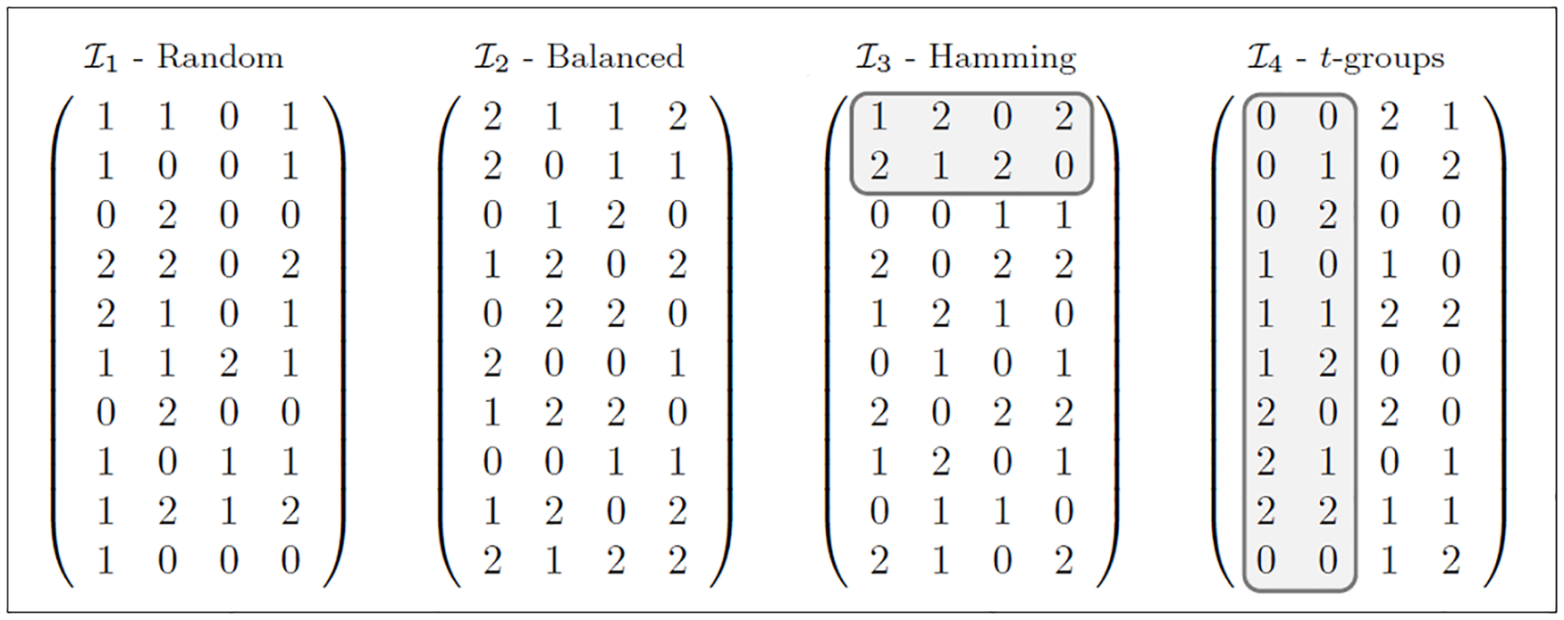
Initialization functions. (a) $I_{1}$ results in 20 missing combinations. (b) $I_{2}$ results in 18 missing combinations. (c) $I_{3}$ results in 15 missing combinations. (d) $I_{4}$ results in 7 missing combinations.

An example of the selection of a row as defined in $I_{3}$ is shown in Fig 2; the matrix C already contains 2 rows, and the third row will be the candidate *d*_1_ because it maximizes the value of $g(d_{j},C)$. Fig 1 (Hamming) shows the full initial matrix.

Finally, the function $I_{4}$ initializes $C_{N\times k}$ based on groups of *t* columns. This function is based on the sub-array $C^{\prime }=CA\left(v^{t};t,t,v\right)$, which is constructed using the *v*^*t*^ combinations of symbols derived from an alphabet of size *v* and a strength value of *t*; e.g., $C^{\prime }=CA\left(3^{2};2,2,3\right)$ will be formed of the elements in the set {00, 01, 02, 10, 11, 12, 20, 21, 22}, where each element represents a row in $C^{\prime }$. The function $I_{4}$ is performed in two steps. In the first step, $C^{\prime }$ is used to define the symbols in the first *t* columns of the matrix C. During this process, juxtaposition of $C^{\prime }$ is applied to complete the *N* rows of C; specifically, $C^{\prime }$ is juxtaposed $\left\lfloor \frac{N}{v^{t}}\right\rfloor v^{t}$ times, and the remaining $N-\left\lfloor \frac{N}{v^{t}}\right\rfloor v^{t}$ rows of C are filled with the first rows of $C^{\prime }$. In the second step, the first *t* columns of C are copied into the next subset of *t* columns whose symbols have not yet been defined, and the values are changed in some pairs of rows; these changes are executed by randomly choosing $\left\lceil \frac{N}{2}\right\rceil$ pairs of rows and, for each pair, exchanging the values of those columns in each row. This step is repeated until all *k* columns of C have been defined. If the number of columns in the last subset (*t*′) is smaller than *t*, then only the first *t*′ columns of $C^{\prime }$ are used. This function is a generalization of the last initialization function presented in [40]. An example of this initialization method is shown in Fig 1 (*t*-groups).

## Algorithms for solving the OSCAR problem

This paper has formally defined the OSCAR problem and has proven that it is NP-complete. Now, various strategies are proposed for solving this problem. This section is devoted to this purpose; throughout the remainder of the section, each proposed approach is described in detail.

Given that a solution to a specific instance of the OSCAR problem is defined by two sets, $J_{R}$ and $J_{C}$, and considering that each of these two sets can be selected using one of three different approaches (exact (E), greedy (G), or meta-heuristic (M)), it is possible to define 9 basic algorithms, as shown in Table 7. The superindices for the EE and GG options indicate the number of variants that have been defined. For the EE approach, the two corresponding algorithms are denoted by $EE_{CR}$ (first the number of columns is reduced, then the number of rows) and $EE_{RC}$ (first the number of rows is reduced, then the number of columns). Three variants have been defined for the GG approach; these variants are denoted by $GG_{CR}$ (first the number of columns is reduced, then the number of rows), $GG_{RC}$ (first the number of rows is reduced, then the number of columns), and $GG_{{}_{C}^{R}}$ (the numbers of columns and rows are reduced in an alternating fashion). Thus, we ultimately present a total of 12 possible algorithms for solving the OSCAR problem.

**Table 7:** Different algorithms for solving the OSCAR problem. The algorithms are grouped by the exact (E), greedy (G), and meta-heuristic (M) approaches.

R	C		
	E	G	M
E	$EE^{2}$	EG	EM
G	GE	$GG^{3}$	GM
M	ME	MG	MM

We first describe the reduction of the numbers of rows and columns of the initial matrix A using the greedy approach. Afterward, the three greedy algorithms $GG_{RC}$, $GG_{CR}$ and $GG_{{}_{C}^{R}}$ are defined. Next, we introduce the exact algorithms $EE_{RC}$ and $EE_{CR}$ for solving the OSCAR problem by exploring the entire search space, which has a size of (NN-δ)(kk-Δ); these algorithms are based on a B&B approach [43]. Next, the meta-heuristic algorithm for solving the OSCAR problem is presented; this algorithm is based on the SA approach. Finally, the six hybrid algorithms GE, EG, GM, MG, ME and EM for solving the OSCAR problem are defined.

### Greedy algorithms $GG_{CR}$, $GG_{RC}$, and $GG_{{}_{C}^{R}}$ for solving the OSCAR problem

**Fig 3:**
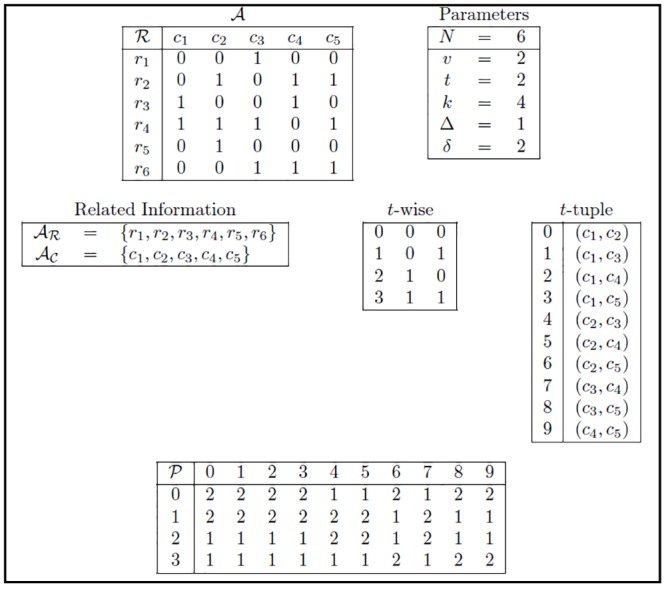
OSCAR instance. Problem instance specified by the matrix A=CA(6;2,5,2) and the values Δ = 1 and *δ* = 2. Related information, *t*-wise combinations, *t*-tuples, and *P* matrix.

The proposed greedy algorithms are based on two functions ($F_{R}$ and $F_{C}$) that reduce the number of rows or columns one element (row or column) at a time, starting from the array A, while considering the number of missing *t*-wise combinations after the reduction process. Examples of all of the greedy strategies proposed in this section are presented based on the OSCAR instance shown in Fig 3. It presents the problem instance specified by the matrix A=CA(6;2,5,2) and the values Δ = 1 and *δ* = 2. It shows each combination of columns, or each *t*-tuple, that is derived from A and all of the possible *t*-wise combinations of symbols that could be found in each of them; it also shows the sets *A*_*R*_ and *A*_*C*_ of rows and columns, respectively. Besides, it shows the auxiliary structure *P*, which is used to store the number of times that each *t*-wise combination is covered in each *t*-tuple; this structure *P* is a matrix of *v*^*t*^ = 2^2^ rows and (kt)=(52)=10 columns, in which each cell *p*_*i*,*j*_ contains the number of times that the *i*^*th*^ *t*-wise combination of symbols appears in A in the subset of columns defined by the *j*^*th*^ *t*-tuple.

#### Greedy approach for reducing the number of rows

The greedy function that reduces the number of rows is denoted by $F_{R}$, and it is defined below. Let $A_{R}=\left\{r_{1},r_{2},...,r_{N}\right\}$ be the set of rows of A, and let $O=\{o_{1},o_{2},\ldots ,o_{N}\}$ be a vector in which each element is associated with a row *r*_*i*_ and has a value equal to the number of *t*-wise combinations that are exclusively covered by the associated row. The function $F_{R}$ selects the row {*r*_*i*_|*i* = min_*j*_{*o*_*j*_}} to be discarded; ties are broken randomly.

The function $F_{R}$ uses the vector O that describes the initial array A to choose a row *r*_*i*_ to be discarded such that the value *o*_*i*_ is minimized. Discarding that row from A results in an array $A^{\prime }$ such that $\tau \left(A^{\prime }\right)=\tau \left(A\right)+o_{i}$, since once row *r*_*i*_ is discarded, the *t*-wise combinations that were covered exclusively by row *r*_*i*_ are no longer covered in $A^{\prime }$. Therefore, the resulting array $A^{\prime }$ without row *r*_*i*_ will be missing the minimum possible number of *t*-wise combinations because *o*_*i*_ has the minimum value among the elements of O. When *o*_*i*_ = 0, row *r*_*i*_ is clearly superfluous, since it does not cover any *t*-wise combinations exclusively.

Every time that a row *r*_*i*_ is discarded in the reduction process, the number of rows that cover each of the *t*-wise combinations covered by the discarded row *r*_*i*_ must be decreased by one. Whenever a *t*-wise combination is then covered by only a single remaining row *j*, the value of *o*_*j*_ must be increased by one. We update the vector O in this way.

The time required to initially populate O for the function $F_{R}$ is O((N+vt+vt(N2))(kt)) (in all algorithms with a greedy component, this process is called getN()), since it is necessary to explore all rows per set of *t* columns, to determine the number of times that each *t*-tuple is covered, and to confirm the *t*-wise combinations that are covered by only one row. The time required to discard a row and update O is O(N+(N-1)(kt)), since it is necessary to first explore the vector O and then, for each set of *t* columns, verify the number of times that each *t*-tuple is covered in at most *N* − 1 rows.

Tables 8, 9 and 10 illustrate the application of getN(A) and $F_{R}\left(O\right)$ to the matrix A defined in Fig 3. The vector O shown in Table 8 is the result of the call to getN(A). Each element of this vector has a value equal to the number of unique *t*-wise combinations covered by the corresponding row; e.g., the value *o*_1_ = 2 implies that row *r*_1_ contains two *t*-wise combinations that are exclusively covered by this row (these are the symbol combinations 00 and 10 corresponding to the *t*-tuples (*c*_2_, *c*_4_) and (*c*_3_, *c*_5_), respectively). Now, a call to $F_{R}\left(O\right)$ will result in an arbitrary selection from among the rows {*r*_1_, *r*_2_, *r*_5_, *r*_6_}; let us assume that *r*_2_ is chosen. The elimination of this row will produce the new vector O shown in Table 10. To illustrate the update operation of $F_{R}$, Table 9 shows how the auxiliary structure *P* is modified in accordance with the *t*-wise combinations that are eliminated with the deletion of row *r*_2_; note that there are 8 new *t*-wise combinations that are now uniquely covered in the remaining rows. In the new vector O, the value of the element corresponding to each of these rows is incremented by the number of *t*-wise combinations in that row for which the corresponding value in *P* has been changed to 1 after the elimination of row *r*_2_. For example, the *t*-wise combinations 01, 00 and 10 associated with *t*-tuples (*c*_1_, *c*_2_), (*c*_1_, *c*_3_), and (*c*_2_, *c*_3_) are newly exclusively covered by row *r*_5_ after the removal of row *r*_2_; consequently, *o*_5_ is increased from 2 to 5 in the new vector O.

**Table 8:** Greedy approach for reducing the number of rows: Examples of the getN() and $F_{R}\left(O\right)$ functions. Results of getN(A).

	*o*_1_	*o*_2_	*o*_3_	*o*_4_	*o*_5_	*o*_6_
O	2	2	6	6	2	2

**Table 9:** Greedy approach for reducing the number of rows: Examples of the getN() and $F_{R}\left(O\right)$ functions. *P* matrix.

*t*-tuple			(*c*_1_, *c*_2_)	(*c*_1_, *c*_3_)	(*c*_1_, *c*_4_)	(*c*_1_, *c*_5_)	(*c*_2_, *c*_3_)	(*c*_2_, *c*_4_)	(*c*_2_, *c*_5_)	(*c*_3_, *c*_4_)	*c*_3_, *c*_5_)	(*c*_4_, *c*_5_)
0	0	0	2	2 → 1	2	2	1	1	2	1	2	2
1	0	1	2 → 1	2	2 → 1	2 → 1	2	2	1	2 → 1	1 → 0	1
2	1	0	1	1	1	1	2 → 1	2	1	2	1	1
3	1	1	1	1	1	1	1	1 → 0	2 → 1	1	2	2 → 1

**Table 10:** Greedy approach for reducing the number of rows: Examples of the getN() and $F_{R}\left(O\right)$ functions. Results of $F_{R}\left(O\right)$.

	*o*_1_	*o*_3_	*o*_4_	*o*_5_	*o*_6_
O	2	7	7	5	5

#### Greedy approach for reducing the number of columns

The function that reduces the number of columns using the greedy approach is denoted by $F_{C}$ and is defined below. Let $A_{C}$ be the set of columns of A; let K, with dimensions of *k* × *k*, be an array in which each element *k*_*i*,*j*_ stores the number of times that columns *i* and *j* together are involved in a missing *t*-wise combination; and let $U=\{u_{1},u_{2},\ldots ,u_{k}\}$ be a vector in which $u_{i}=\sum_{j=1}^{k}k_{i,j}$. The function $F_{C}$ selects the column {*c*_*i*_|*i* = max_*j*_{*u*_*j*_}} to be discarded; ties are broken randomly. Whenever a column *i* is discarded, the vector U is updated by subtracting the value *k*_*i*,*j*_ from *u*_*j*_ for all *j* ≠ *i*. Each element in U is associated with a column, and its value is equal to the number of times that column is involved in a missing *t*-wise combination.

In summary, the function $F_{C}$ chooses a column *i* associated with the maximum value *u*_*i*_ in the vector U. When discarding column *i*, we obtain an array $A^{\prime }$ such that $\tau \left(A^{\prime }\right)=\sum_{j=0}^{k-1}u_{j}$, since once column *i* has been discarded, the associated missing combinations involving column *i* are deleted. Therefore, the resulting array $A^{\prime }$ will have the minimum number of missing *t*-wise combinations, since *u*_*i*_ has the greatest value among the elements of U.

When we discard a column, the values of the elements of U must be updated. To do so, a value of −1 is assigned to *u*_*i*_, and the value of each element *u*_*j*_ such that *j* ≠ *i* is updated based on its interaction with the recently discarded column; i.e., *u*_*j*_ = *u*_*j*_ − *k*_*i*,*j*_. This process is intuitively illustrated as follows. Suppose that we have a set of *n* criminals who are accused of having committed *m* crimes together, and suppose that the authorities have found that a certain criminal *s* is the only one who committed *l* of these crimes, where *l* ≤ *m*; then, the number of crimes of which each of the remaining criminals is accused must be decreased in accordance with his initially suspected degree of participation in committing crimes with criminal *s*.

The time required to initially populate U and K for the function $F_{C}$ is O((N+(t2)+t)(kt)) (in all algorithms with a greedy component, this process is called getK()), since for each set of *t* columns, all *N* rows must be explored, the vector G must then be updated based on the missing *t*-wise combinations in these columns, and K must be updated for all possible pairs in this set of *t* columns. The time required to discard a column and update U and K accordingly is *O*(2*k*), since the vector U must be explored to obtain the column *i* with the greatest value, and column *i* of K must then be explored to update U.

Tables 11 and 12 illustrate the application of getk(A) and $F_{C}\left(K,k,U\right)$ to the matrix A defined in Fig 3. The matrix K and the vector U shown in Table 11 are the results of the call to getk(A); given that the initial matrix A is a CA, all values in K and U are zero because there are no missing *t*-wise combinations. Now, a call to $F_{C}\left(K,k,U\right)$ will result in the arbitrary selection of a column from among {*c*_1_, *c*_2_, *c*_3_, *c*_4_, *c*_5_}; let us assume that *c*_2_ is chosen. The elimination of this column will produce the new vector U shown in Table 12, which also has zero missing *t*-wise combinations because the values *c*_*j*_, for *j* ≠ 2, are all zero.

**Table 11:** Greedy approach for reducing the number of columns: Examples of the getk(A) and $F_{C}\left(K,k,U\right)$ functions.

R	K					U	
	*c*_1_	*c*_2_	*c*_3_	*c*_4_	*c*_5_	*u*_1_	0
*c*_1_	-	0	0	0	0	*u*_2_	0
*c*_2_	0	-	0	0	0	*u*_3_	0
*c*_3_	0	0	-	0	0	*u*_4_	0
*c*_4_	0	0	0	-	0	*u*_5_	0
*c*_5_	0	0	0	0	-		

**Table 12:** Greedy approach for reducing the number of columns: New vector U.

	*u*_1_	*u*_3_	*u*_4_	*u*_5_
*U*_*update*_	0	0	0	0

Now that the greedy functions $F_{R}$ and $F_{C}$ have been defined, the three greedy algorithms are introduced below.

#### Greedy algorithm $GG_{CR}$

$GG_{CR}$ is a greedy algorithm that first reduces A to a matrix with *k* − Δ columns using the function $F_{C}$. The newly formed array is denoted by $A^{\prime }$ and has *N* rows and *k* − Δ columns. Then, $A^{\prime }$ is further reduced to a matrix with *N* − *δ* rows using the function $F_{R}$, yielding the solution B. Algorithm 1 describes the $GG_{CR}$ approach.

**Algorithm 1**

1: **function** $GG_{CR}\left(A,\delta ,\Delta \right)$

2:  $J_{R}\;\leftarrow \;\emptyset$

3:  $J_{C}\;\leftarrow \;\emptyset$

4:  getK(A)

5:  **for** *i* < Δ **do**

6:   $c\;\leftarrow \;F_{C}\left(K,k,U\right)$

7:   $J_{C}\;\leftarrow \;J_{C}+c$

8:  **end for**

9:  $A^{\prime }\;\leftarrow \;A_{C}-J_{C}$

10:  getN$\left(A^{\prime }\right)$

11:  **for** *i* < *δ* **do**

12:   $r\;\leftarrow \;F_{R}(O)$

13:   $J_{R}\;\leftarrow \;J_{R}+r$

14:  **end for**

15:  $A^{\prime }\;\leftarrow \;A_{R}-J_{R}$

16:  $B^{\prime }\;\leftarrow \;A^{\prime }$

17:  **return** (c,B)

18: **end function**

The time required to execute $GG_{CR}$ can be calculated from the times required for populating and updating the necessary structures, as follows: O((N+(t2)+t)(kt)+Δ(2k)+(N+vt+vt(N2))(k-Δt)+δ(N+(N-1)(k-Δt))).

During the execution of this algorithm, Δ columns are first discarded from A following the defined reduction process, resulting in an array $A^{\prime }$ with *k* − Δ columns; then, the necessary structures for eliminating rows from $A^{\prime }$ are populated, and finally, *δ* rows are discarded following the defined reduction process, yielding the solution B.

**Fig 4:**
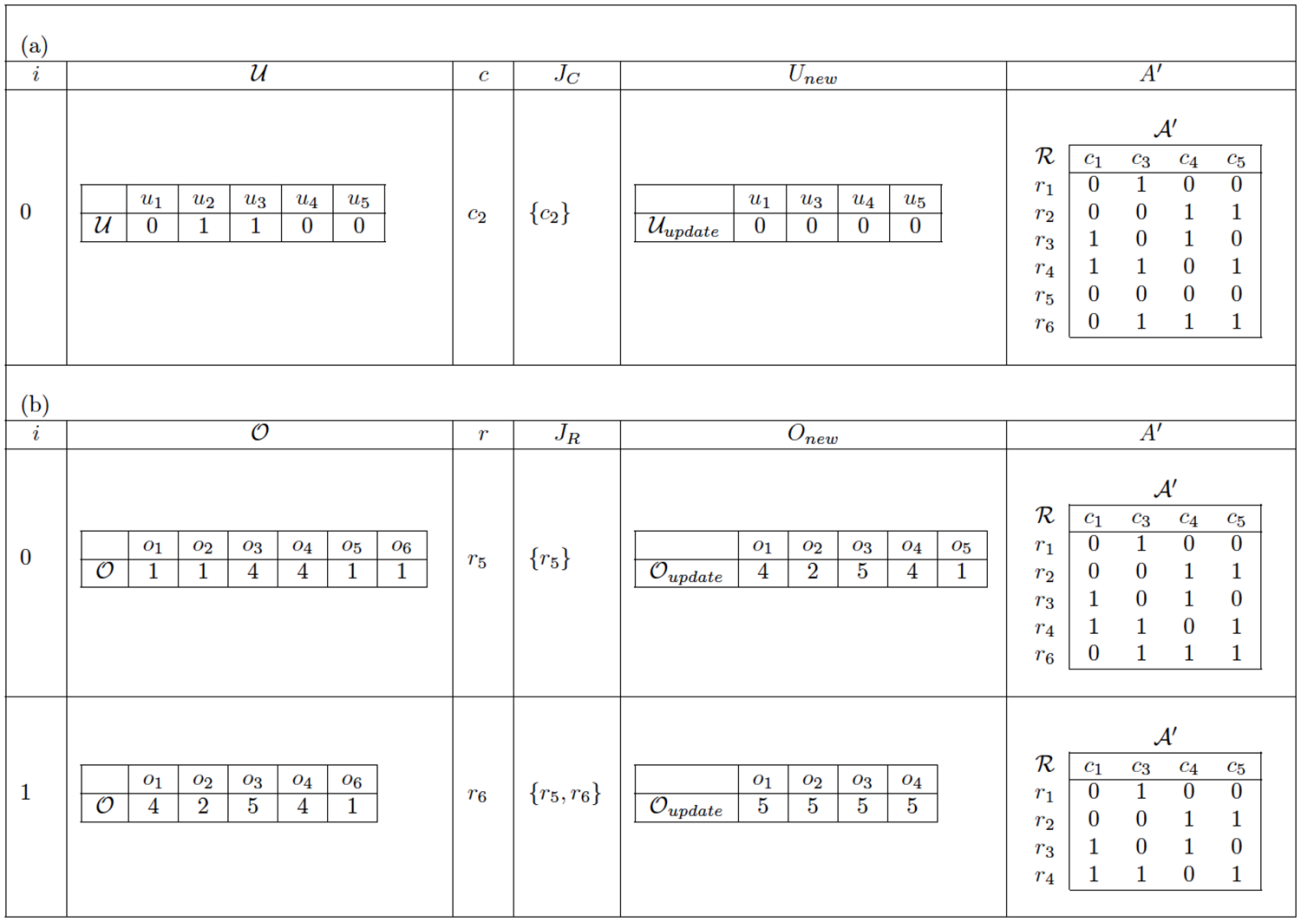
Example of $GG_{CR}$. (a) Discarding columns. (b) Discarding rows.

Fig 4 shows an example of the application of $GG_{CR}$ to the problem instance presented in Fig 3. This table illustrates how the initial matrix A evolves into the final matrix B. First, Fig 4(a) shows the changes made to A due to the elimination of columns (see the loop in lines 5 to 8); for each iteration *i* of this loop, the table presents the initial vector U, the set *J*_*C*_ of columns chosen so far, the vector $U_{new}$ obtained by updating U after the elimination of the column *c* selected in that iteration, and the resulting matrix $A^{\prime }$ after that iteration. This part of the algorithm is performed only once because Δ = 1. Subsequently, Fig 4(b) presents the changes made to the last matrix $A^{\prime }$ obtained in the previous process due to the elimination of rows (see the loop in lines 11 to 14); for each iteration *i* of this loop, the table presents the vector O derived from the previous matrix $A^{\prime }$, the set *J*_*R*_ of rows chosen so far, the updated vector $O_{new}$ obtained after the elimination of the row *r* selected in that iteration, and the resulting matrix $A^{\prime }$ after that iteration. This second loop is repeated twice because *δ* = 2. The last matrix $A^{\prime }$ obtained in the second part of $GG_{CR}$ is returned as the final matrix B.

#### Greedy algorithm $GG_{RC}$

The greedy algorithm $GG_{RC}$ first removes *δ* rows from A using the function $F_{R}$ to obtain an array $A^{\prime }$ with *N* − *δ* rows and *k* columns. Then, $A^{\prime }$ is reduced to a matrix with *k* − Δ columns using the function $F_{C}$ to obtain the final solution B. Algorithm 2 describes the $GG_{RC}$ approach.

**Algorithm 2**

1: **function** $GG_{RC}\left(A,\delta ,\Delta \right)$

2:  $J_{R}=\emptyset$

3:  $J_{C}=\emptyset$

4:  getN(A)

5:  **for** *i* < *δ* **do**

6:   $r\;\leftarrow \;F_{R}(O)$

7:   $J_{R}=J_{R}+r$

8:  **end for**

9:  $A^{\prime }\;\leftarrow \;A_{R}-J_{R}$

10:  getK$\left(A^{\prime }\right)$

11:  **for** *i* < Δ **do**

12:   $c\;\leftarrow \;F_{C}(K,k,U)$

13:   $J_{C}\;\leftarrow \;J_{C}+c$

14:  **end for**

15:  $A^{\prime }\;\leftarrow \;A_{C}-J_{C}$

16:  $B\;\leftarrow \;A^{\prime }$

17:  **return** (c,B)

18: **end function**

The time required to execute $GG_{RC}$ can be calculated from the times required for populating and updating the necessary structures. The result is O((N+vt+vt(N2))(kt)+δ(N+N-1(kt))+(N+(t2))(kt))+Δ(2k)), since *N* − *δ* rows are first discarded from the input array A, generating an array $A^{\prime }$ with *N* − *δ* rows and *k* columns, and this array is then reduced to one with *k* − Δ columns to obtain the solution B.

**Fig 5:**
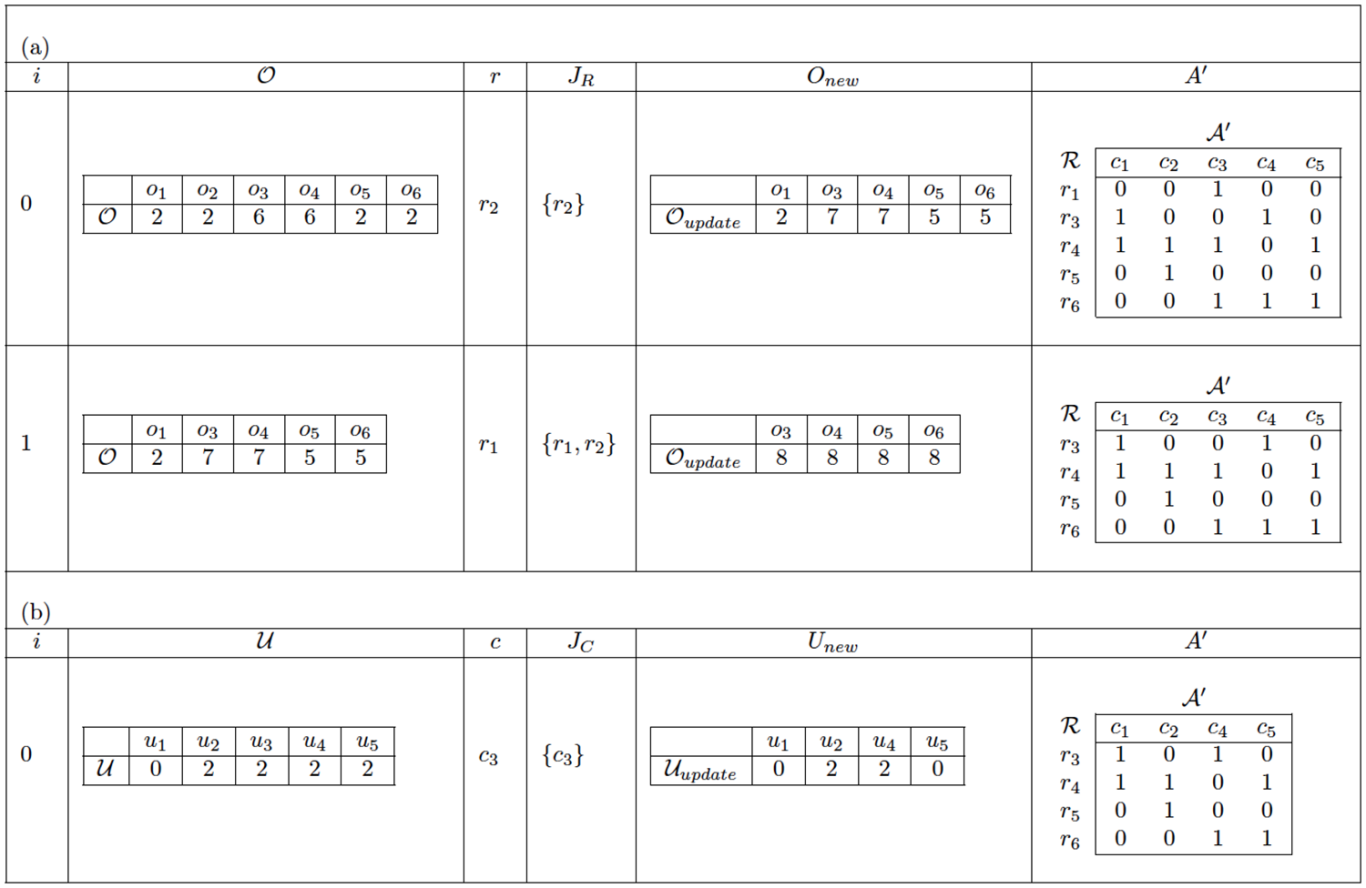
Example of $GG_{RC}$. (a) Discarding rows. (b) Discarding columns.

Fig 5 shows an example of the application of $GG_{RC}$ to the problem instance presented in Fig 3. This table illustrates how the initial matrix A evolves into the final matrix B. First, Fig 5(a) presents the changes made to A due to the elimination of rows (see the loop in lines 5 to 8); for each iteration *i* of this loop, the table presents the initial vector O, the set *J*_*R*_ of rows chosen so far, the vector $O_{new}$ obtained by updating O after the elimination of the row *r* selected in that iteration, and the resulting matrix $A^{\prime }$ after that iteration. This part of the algorithm is repeated twice because *δ* = 2. Subsequently, Fig 5(b) presents the changes made to the last matrix $A^{\prime }$ obtained in the previous process due to the elimination of columns (see the loop in lines 11 to 14); for each iteration *i* of this loop, the table presents the vector U derived from the last matrix $A^{\prime }$, the set *J*_*C*_ of columns chosen so far, the updated vector $U_{new}$ after the elimination of the column *c* selected in that iteration, and the resulting matrix $A^{\prime }$ after that iteration. This second loop is executed only once because Δ = 1. The last matrix $A^{\prime }$ obtained in the second part of $GG_{RC}$ is returned as the final matrix B.

#### Greedy algorithm $GG_{{}_{C}^{R}}$

The greedy algorithm $GG_{{}_{C}^{R}}$ distributes the elimination of Δ columns and *δ* rows in a round-robin fashion. This algorithm alternately discards first a single row and then some number of columns until the number of rows has been reduced to *N* − *δ*. This algorithm uses a vector D with *δ* elements, where each element *d*_*i*_, corresponding to the *i*^*th*^ discarded row, indicates the number of columns that should be discarded immediately after discarding that row. When Δ > *δ*, the first *δ* − 1 elements of D are each filled with a value of $\left\lfloor \frac{\Delta }{\delta }\right\rfloor$, and the last one is filled with a value of $\left\lceil \frac{\Delta }{\delta }\right\rceil$. When *δ* ≥ Δ, the first Δ elements of D are each filled with a value of one. Once the Δ columns have been distributed among the *δ* rows, one row is discarded, and then, the number of columns is reduced to *k* − *d*_*i*_. Hence, with the exploration of each element *i* of the vector D, the numbers of rows and columns of the array $A^{\prime }$ will be decreased to *N*_*i*_ = *N* − *i* and $k_{i}=k-\sum_{j=1}^{i-1}d_{j}$, respectively. Algorithm 3 describes the $GG_{{}_{C}^{R}}$ approach.

The time required to execute $GG_{{}_{C}^{R}}$ can be obtained by considering how the numbers of columns and rows of $A^{\prime }$ will be reduced while exploring the vector D; the result is O(∑i=1δ(Ni+vt+(vt(kit)))(kit)+(Ni+(Ni-1(kit))+(Ni+(t2)+t)(kit)+di(2(ki)). As each element *i* of D is explored, first, the necessary structures are populated to discard rows from the array $A^{\prime }$, which has *N* − *i* + 1 rows and $k-\sum_{j=1}^{i-1}d_{j}$ columns, and the number of rows is reduced to *N* − 1. Next, it is necessary to populate the structures needed to discard columns from the new array $A^{\prime }$ with the reduced number of rows, and then, the number of columns is reduced to *k* − *d*_*i*_.

**Fig 6:**
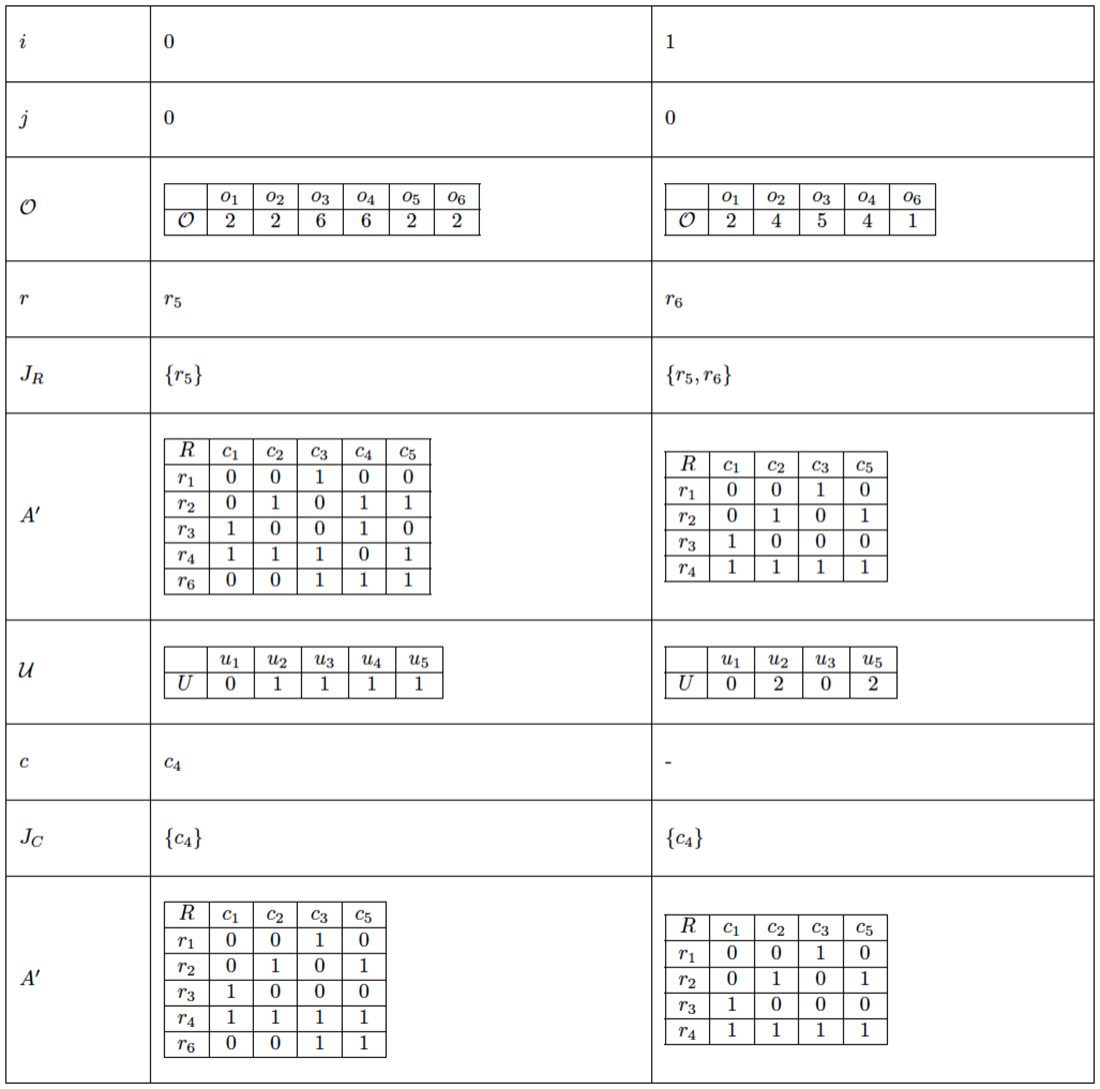
Example of $GG_{{}_{C}^{R}}$. Column 1 shows the main structures used throughout the algorithm, and each of the remaining columns represents a different iteration of the main algorithm.

Fig 6 shows an example of the application of $GG_{{}_{C}^{R}}$ to the problem instance presented in Fig 3. Because Δ is not greater than *δ* in this instance, the number of columns that must be eliminated with the elimination of each row is given by the vector $D=\{d_{1}=1,d_{2}=0\}$; i.e., after the deletion of the first row, one column must be deleted, and then the algorithm proceeds to the deletion of the second row to satisfy the value *δ* = 2. The example shown in Fig 6 illustrates the reduction process for the instance given in Fig 3. The first column lists the main structures that are changed during the execution of the algorithm. Each of the remaining columns in Fig 6 represents a different iteration of the main loop of the algorithm.

**Algorithm 3**

1: **function** $GG_{{}_{C}^{R}}\left(A,\delta ,\Delta \right)$

2:  $J_{R}=\emptyset$

3:  $J_{C}=\emptyset$

4:  **if** Δ > 0 **then**

5:   **if** Δ > *δ* **then**

6:    **for** *i* ← 0 **to** *i* < *δ* − 1 **do**

7:     $d_{i}\leftarrow \lfloor \frac{\Delta }{\delta }\rfloor$

8:    **end for**

9:    $d_{\delta -1}\leftarrow \lceil \frac{\Delta }{\delta }\rceil$

10:   **else**

11:    **for** *i* ← 0 **to** *i* < Δ **do**

12:     *d*_*i*_ ← 1

13:    **end for**

14:   **end if**

15:   **for** *i* ← 0 **to** *i* < *δ* **do**

16:    getN$\left(A^{\prime }\right)$

17:    $r\leftarrow F_{R}(O)$

18:    $A^{\prime }\leftarrow A_{R}^{\prime }-r$

19:    getK$\left(A^{\prime }\right)$

20:    **for** *j* ← 0 **to** *j* < *d*_*i*_ **do**

21:     $c\leftarrow F_{C}(K,k,U)$

22:     $A^{\prime }\leftarrow A_{C}^{\prime }-c$

23:    **end for**

24:   **end for**

25:   $B\leftarrow A^{\prime }$

26:  **else**

27:   $GG_{RC}\left(A,\delta ,\Delta \right)$

28:  **end if**

29:  **return** (B)

30: **end function**

### Meta-heuristic algorithm MM

The approximate algorithm MM for searching for a solution to the OSCAR problem is based on the SA approach and is described in Algorithm 4. This approach is a general-purpose stochastic optimization strategy that has been proven to be an efficient means of approximating global optimal solutions to many NP-complete combinatorial optimization problems. In this strategy, a solution $W_{u}$ is first constructed using the Initialize(…) method, and this solution is designated as the first global best solution $W^{*}$; then, the algorithm enters an iterative improvement process, controlled by the length of the Markov chain, until a certain termination criterion is achieved. In each iteration of this improvement process, a new solution $W_{v}$ is generated using the GenerateNeighbor(…) method, and this new solution is substituted for $W_{u}$ whenever its quality is superior to that of the current solution or the probability condition is satisfied. The probability condition is based on the Boltzmann distribution, and it is defined with respect to the values of an initial temperature $T_{i}$, a final temperature $T_{f}$, and a quality function *τ*(…) of $W_{u}$ and $W_{v}$. The global best $W^{*}$ is also updated every time a solution $W_{v}$ improves upon it. The details of these procedures are presented in the remainder of this subsection.

**Algorithm 4**

1: **function** $MM\left(A,\delta ,\Delta ,T_{i},T_{f},\alpha ,E,P\right)$

2:  $J_{R}=\emptyset$

3:  $J_{C}=\emptyset$

4:  $W_{v}$ ← Initialize(*N*, *k*, *δ*, Δ)

5:  $W_{u}\leftarrow W_{v}$

6:  $W^{*}\leftarrow W_{v}$

7:  *L* ← (*N* + *k*)*v*

8:  *n* ← 0

9:  **while** n<E **and** $T_{i}<T_{f}$ **do**

10:   **for** *i* ← 0 **to** *i* < *L* **do**

11:    $W_{v}$ ← GenerateNeighbor $\left(S,W_{u},P\right)$

12:    **if** $\tau \left(W_{v}\right)\leq \tau \left(W_{u}\right)$ **then**

13:     $W_{u}\leftarrow W_{v}$

14:     **if** $\tau \left(W_{u}\right)\leq \tau \left(W^{*}\right)$ **then**

15:      $W^{*}\leftarrow W_{u}$

16:     **else if** random(0…1) $\leq e^{\frac{\tau \left(W_{v}\right)-\tau \left(W_{u}\right)}{T_{i}}}$ **then**

17:      $W_{u}\leftarrow W_{v}$

18:     **end if**

19:    **end if**

20:   **end for**

21:   $T_{i}\leftarrow \alpha *T_{i}$

22:   *n ← n* + 1

23:  **end while**

24:  B ← constructMatrix $\left(W^{*}\right)$

25:  **return** (B)

26: **end function**

A solution is represented by a vector W with *N* + *k* elements, which identify the subsets of the rows and columns of the matrix A that are used to construct a new submatrix B. All of the elements are binary; each of the first *N* elements is associated with a particular row in A, and each of the last *k* elements is associated with one of its columns. The subset $J_{R}\subset A_{R}$ of rows to be excluded from the new submatrix B consists of the rows of $A_{R}$ that are associated with the corresponding elements in W that have a value of 1. Similarly, the subset $J_{C}\subset A_{C}$ of columns to be excluded from the new submatrix B consists of the columns of $A_{C}$ that are associated with the corresponding elements in W that have a value of 1.

The Initialize(N, k, *δ*, Δ) method is used to construct the first solution $W_{u}$ in the proposed strategy. This method uses the best greedy algorithm among those proposed in this paper. The greedy strategy is chosen based on preliminary experiments for *SA*_*OSCAR*_.

Once the initial solution $W_{u}$ has been created, it is modified using the neighborhood function $\text{GenerateNeighbor}(S,W_{u},P)$. This method randomly chooses from among three predefined strategies, $S_{1}$, $S_{2}$ and $S_{3}$, to create a new solution $W_{v}$. In the strategy $S_{1}$, a row of $W_{u}$ is exchanged; one existing row in the solution is randomly removed and replaced with a different row not previously included. The strategy $S_{2}$ follows the same approach as that of $S_{1}$ but for columns. Finally, the strategy $S_{3}$ is the combination of the previous two. The use of the neighborhood function is controlled by the parameter *L*, which is called the Markov chain length.

The quality function, or evaluation function, that is used to measure the fitness of a solution is derived from the definition of the OSCAR problem. This function, denoted by τ(W), counts the number of missing *t*-wise combinations. We note that one missing *t*-wise combination represents a *t*-tuple for a particular combination of columns that the matrix does not contain but would be required to cover in order for the matrix to be a *CA*(*N* − *δ*; *t*, *k* − Δ, *v*).

Finally, the cooling schedule is controlled by a cooling factor *α*, which is used to gradually decrease an initial temperature $T_{i}$ until it reaches a given final temperature $T_{f}$, marking the end of the algorithm. Note that the algorithm also includes an alternative termination criterion, which is defined as a maximum number of iterations of the main loop.

The time required to execute MM is *O*(*iL*), where $i=\frac{\log_{\alpha }T_{f}-\log_{\alpha }T_{i}}{\log_{\alpha }\alpha }$ is the number of temperature decrements necessary to reach $T_{f}$.

### Exact algorithms $EE_{RC}$ and $EE_{CR}$

The exact algorithms $EE_{RC}$ and $EE_{CR}$ were previously reported in [24]. They follow the B&B strategy (cf. [43]) and avoid the need to explore the entire feasible region to find the optimal solution. The general idea behind these algorithms is described in Algorithm 5 and Algorithm 6.

These previously presented algorithms first construct an initial solution B using a greedy strategy; then, they remove from this solution all possible combinations of rows and columns such that the resulting matrix $B^{\prime }$ has *N* − *δ* rows and *k* − Δ columns. Because the order in which the rows and columns are removed matters, the strategies differ in their selection of which elements are removed first. Whereas $EE_{RC}$ first removes a subset of columns, $EE_{CR}$ first removes a subset of rows. Both algorithms, after the first selected elements have been removed, perform a B&B search over the columns and/or rows, testing each element one by one, in order to find the submatrix $B^{*}$ with the minimum number of missing *t*-wise combinations.

**Algorithm 5**

1: **function** $EE_{RC}$ (A,δ,Δ)

2:   $B^{*}$ ← bestGreedy (A,δ,Δ)

3:   $B\leftarrow B^{*}$

4:   UpperBound←τ(B)

5:   **for each** $J_{C}\subset A_{C}$ **and** $|J_{C}|=\Delta$ **do**

6:    $B^{\prime }$ ← Eliminate $\left(B,J_{C}\right)$

7:   **for each** $J_{R}\subset A_{R}\;\text{and}\;\left|J_{R}\right|=\delta$ **do**

8:     $B^{\prime \prime }$ ← Eliminate $\left(B^{\prime },J_{R}\right)$

9:    **if** $\tau (B^{\prime \prime })<UpperBound$ **then**

10:      $B^{*}\leftarrow B^{\prime \prime }$

11:      $UpperBound\leftarrow \tau (B^{\prime \prime })$

12:    **end if**

13:   **end for**

14:  **end for**

15:  **return** $\left(B^{*}\right)$

16: **end function**

**Algorithm 6**

1: **function** $EE_{CR}(A,\delta ,\Delta )$

2:   $B^{*}$ ← bestGreedy (A,δ,Δ)

3:   $B\leftarrow B^{*}$

4:   UpperBound←τ(B)

5:   **for each** $J_{R}\subset A_{R}$ **and** $|J_{R}|=\delta$ **do**

6:    $B^{\prime }$ ← Eliminate $\left(B,J_{R}\right)$

7:    **for each** $J_{C}\subset A_{C}$ **and** $|J_{C}|=\Delta$ **do**

8:     $B^{\prime \prime }$ ← Eliminate $\left(B^{\prime },J_{C}\right)$

9:     **if** $\tau (B^{\prime \prime })<UpperBound$ **then**

10:      $B^{*}\leftarrow B^{\prime \prime }$

11:      $UpperBound\leftarrow \tau (B^{\prime \prime })$

12:    **end if**

13:   **end for**

14:  **end for**

15:  **return** $\left(B^{*}\right)$

16: **end function**

We note that the elimination of the second set of elements obeys a lexicographical order given by the columns or rows that are being deleted. Moreover, during the search process, both algorithms avoid the elimination of certain columns or rows that would exert undesirable effects on the final submatrix (i.e., selections for which the number of missing *t*-wise combinations would increase over a certain upper bound); for a more detailed description of the algorithms, refer to [24]. The time required to execute either $EE_{CR}$ or $EE_{RC}$ is O((NN-δ)(kk-Δ)) since, in the worst case, it is not possible to discard solutions.

### Hybrid algorithms for solving the OSCAR problem

#### Hybrid algorithm GE

The algorithm GE combines the greedy and exact approaches to solve the OSCAR problem. The algorithm proceeds in two phases. First, it chooses a set of Δ columns and removes them from the initial matrix A; the resulting matrix is denoted by $A^{\prime }$ and has dimensions of *N* × (*k* − Δ). Afterward, the algorithm discards *δ* rows from $A^{\prime }$ in a greedy manner to construct a possible solution $B^{\prime }$, i.e., a matrix with dimensions of (*N* − *δ*) × (*k* − Δ), for the OSCAR instance at hand. To obtain the best solution B, the algorithm explores all possible combinations of Δ columns and identifies the best matrix B from among all matrices $B^{\prime }$ constructed during the process described above.

**Algorithm 7**

1: **function** GE (A,δ,Δ)

2:  **for** *i* ← 0 **to** i<(kk-Δ) **do**

3:    $J_{C}$ ← GREATERTHANPOLYNOMIAL $\left(J_{C}\right)$

4:    $A^{\prime }\leftarrow A-J_{C}^{c}$

5:    getN $\left(A^{\prime }\right)$

6:    $J_{R}=\emptyset$

7:   **for** *j* ← 0 **to** *j* < *δ* **do**

8:     $r\leftarrow F_{R}(O)$

9:     $J_{R}^{c}=J_{R}^{c}+r$

10:    **end for**

11:    $B^{\prime }\leftarrow A_{R}^{\prime }-J_{R}^{c}$

12:    **if** $\tau \left(B^{\prime }\right)<\tau \left(B\right)$ **then**

13:     $B\leftarrow B^{\prime }$

14:     $\tau \left(B\right)\leftarrow \tau \left(B^{\prime }\right)$

15:   **end if**

16:  **end for**

17:  **return** (B)

18: **end function**

The algorithm GE for solving the OSCAR problem is described in Algorithm 7. Each combination of *k* − Δ columns is represented by the vector $J_{C}$. Each new combination of *k* − Δ columns is computed by the function GREATERTHANPOLYNOMIAL() [44]. An array $A^{\prime }$, with dimensions of *N* × (*k* − Δ), is constructed using the columns indicated by $J_{C}$. Then, the algorithm populates the necessary structures to reduce $A^{\prime }$ to a matrix with *N* − *δ* rows, and this reduction process yields an array $B^{\prime }$. The best solution that has been found so far during the exploration process is represented by B. Whenever $\tau \left(B^{\prime }\right)<\tau \left(B\right)$ for a newly constructed matrix $B^{\prime }$, the matrix B is replaced with $B^{\prime }$.

The time required to execute GE can be derived from the times required for populating the necessary structures, reducing the number of rows, and updating the necessary values. This time is proportional to O((kk-Δ)((N+vt+vt(N2))(k-Δt)+δ(N+(N-1)(k-Δt)))).

#### Hybrid algorithm EG

The algorithm EG also combines the exact and greedy approaches to find a solution to the OSCAR problem; compared with GE, the difference is that it explores all possible combinations of *δ* rows that can be eliminated from the original matrix. The algorithm proceeds in two phases. First, it chooses a set of *δ* rows and removes them from the initial matrix A; the resulting matrix is denoted by $A^{\prime }$ and has dimensions of (*N* − *δ*) × *k*. Subsequently, the algorithm greedily discards Δ columns from $A^{\prime }$ to construct a possible solution $B^{\prime }$, i.e., a matrix with dimensions of (*N* − *δ*) × (*k* − Δ), for the OSCAR instance at hand. To obtain the best solution B, the algorithm explores all possible combinations of *δ* rows and identifies the best matrix B from among all matrices $B^{\prime }$ constructed during the process described above.

The algorithm EG for solving the OSCAR problem is described in Algorithm 8. Each combination of *N* − *δ* rows is represented by the vector $J_{R}$. Each new combination of *N* − *δ* rows is computed by the function GREATERTHANPOLYNOMIAL(). An array $A^{\prime }$, with dimensions of (*N* − *δ*) × *k*, is constructed using the rows indicated by $J_{R}$. Then, the algorithm populates the necessary structures to greedily reduce $A^{\prime }$ to a matrix with *k* − Δ columns, and this reduction process yields an array $B^{\prime }$. The best solution that has been found so far during the exploration process is represented by B. Whenever $\tau \left(B^{\prime }\right)<\tau \left(B\right)$ for a newly constructed matrix $B^{\prime }$, the matrix B is replaced with $B^{\prime }$.

**Algorithm 8**

1: **function** EG(A,δ,Δ)

2:  **for** *i* ← 0 **to** i<(NN-δ) **do**

3:    $J_{R}$ ← GREATERTHANPOLYNOMIAL$\left(J_{R}\right)$

4:    $A^{\prime }\;\leftarrow \;A-{J_{R}}^{c}$

5:   getK$\left(A^{\prime }\right)$

6:   $J_{C}=\emptyset$

7:   **for** *j* ← 0 **to** *j* < Δ **do**

8:     $c\;\leftarrow \;F_{C}\left(G,K\right)$

9:     ${J_{C}}^{c}\;\leftarrow \;{J_{C}}^{c}+c$

10:   **end for**

11:   $B^{\prime }\;\leftarrow \;{A_{C}}^{\prime }-{J_{C}}^{c}$

12:   **if** $\tau \left(B^{\prime }\right)<\tau \left(B\right)$ **then**

13:     $B\;\leftarrow \;B^{\prime }$

14:     $\tau (B)\;\leftarrow \;\tau \left(B^{\prime }\right)$

15:   **end if**

16:  **end for**

17:  **return** (B)

18: **end function**

The time required to execute EG can be derived from the times required for populating the necessary structures, reducing the number of columns, and updating the necessary values. This time is proportional to O((NN-δ)((N+(t2)+t)(kt)+Δ(2k))).

#### Hybrid algorithm GM

The algorithm GM uses a hybrid strategy that combines the SA meta-heuristic [45] with the greedy approach to construct a solution to the OSCAR problem. In each iteration of GM, a local search is performed over the possible set of columns that can be eliminated to obtain a matrix $A^{\prime }$ with dimensions of *N* × (*k* − Δ). Afterward, the matrix $A^{\prime }$ is subjected to a greedy process to reduce its size by *δ* rows and thus to construct a solution B with dimensions of (*N* − *δ*) × (*k* − Δ) for the OSCAR instance at hand. Once the matrix B has been built, the Boltzmann criterion is used as usual in SA. The details of the strategy are presented in the remainder of this subsection.

Algorithm 9 describes the proposed GM approach for solving the OSCAR problem. The algorithm GM uses a vector *W*_*u*_ of size *k* to represent the state of each column in the solution B. The elements of the vector take values of *w*_*i*_ ∈ {0, 1} for 1 ≤ *i* ≤ *k*, where a value of 0 indicates that the corresponding column is not present in the solution and a value of 1 indicates otherwise. In addition, two constraints are imposed to obtain a proper OSCAR solution: there must be *k* − Δ elements with a value of *w*_*i*_ = 1, and there must be Δ elements with a value of *w*_*i*_ = 0.

**Algorithm 9**

1: **function** $GM\left(A,\delta ,\Delta ,T_{i},T_{f},\alpha ,E\right)$

2:   $W_{v}$ ← Initialize(*k*, Δ)

3:   $W_{u}\;\leftarrow \;W_{v}$

4:  $W^{\prime }\;\leftarrow \;W_{v}$

5:  *L* ← (*N* + *k*)*v*

6:  *n* ← 0

7:  **while** $n<E\;\;\text{and}\;\;T_{i}<T_{f}$ **do**

8:   **for** *i* ← 0 **to** *i* < *L* **do**

9:     $W_{v}$ ← generateNeighbor$\left(W_{u}\right)$

10:    $A^{\prime }$ ← ConstructSolution

11:    getN$\left(A^{\prime }\right)$

12:    **for** *i* ← 0 **to** *i* < *δ* **do**

13:      $F_{R}(O)$

14:    **end for**

15:    **if** $\tau \left(W_{v}\right)\leq \tau \left(W_{u}\right)$ **then**

16:     $W_{u}\;\leftarrow \;W_{v}$

17:     **if** $\tau \left(W_{u}\right)\leq \tau \left(W^{\prime }\right)$ **then**

18:       $W^{\prime }\;\leftarrow \;W_{u}$

19:     **end if**

20:    **else if** random(0 … 1) $\leq e^{\frac{\tau \left(W_{v}\right)-\tau \left(W_{u}\right)}{T_{i}}}$ **then**

21:      $W_{u}\;\leftarrow \;W_{v}$

22:    **end if**

23:   **end for**

24:   $T_{i}\;\leftarrow \;\alpha \cdot T_{i}$

25:  **end while**

26:  B ← constructMatrix$\left(W^{\prime }\right)$

27:  **return** (B)

28: **end function**

The algorithm GM uses a set of perturbations to the vector $W_{u}$ as its neighborhood function. For this purpose, it chooses two elements *w*_*i*_ and *w*_*j*_, where *w*_*i*_ ≠ *w*_*j*_, and interchanges their values. The new solution formed via this perturbation, which is a neighbor of $W_{u}$, is denoted by $W_{v}$.

Finally, the evaluation function used in GM is *τ*, the number of missing combinations in a created matrix. This function is also used to evaluate the matrices created during the local search.

The time required to execute GM is proportional to *O*(*iLF*_*R*_), where $i=\frac{\log_{\alpha }\;T_{f}-\log_{\alpha }\;T_{i}}{\log_{\alpha }\;\alpha }$ is the number of temperature decrements necessary to reach $T_{f}$ and *F*_*R*_ is the time cost of the greedy approach for eliminating rows.

#### Hybrid algorithm MG

The algorithm MG uses another hybrid strategy that combines the SA meta-heuristic [45] with the greedy approach to construct a solution to the OSCAR problem. In each iteration of MG, a local search is performed over the possible set of rows that can be eliminated to obtain a matrix $A^{\prime }$ with dimensions of (*N* − *δ*) × *k*. Afterward, the matrix $A^{\prime }$ is subjected to a greedy process to reduce its size by Δ columns and thus to construct a solution B with dimensions of (*N* − *δ*) × (*k* − Δ) for the OSCAR instance at hand. Once the matrix B has been built, the Boltzmann criterion is used as usual in SA. The details of the strategy are presented in the remainder of this subsection.

Algorithm 10 describes the proposed MG approach for solving the OSCAR problem. The algorithm MG uses a vector *W*_*u*_ of size *N* to represent the state of each row in the solution B. The elements of the vector take values of *w*_*i*_ ∈ {0, 1} for 1 ≤ *i* ≤ *k*, where a value of 0 indicates that the corresponding row is not present in the solution and a value of 1 indicates otherwise. The constraints imposed to ensure a proper OSCAR solution are as follows: there must be *N* − *δ* elements with a value of *w*_*i*_ = 1 and *δ* elements with a value of *w*_*i*_ = 0.

**Algorithm 10**

1: **function** $MG\left(A_{N\times k},t,\delta ,\Delta ,T_{i},T_{f},\alpha ,E\right)$

2:  $W_{v}$ ← Initialize(*k*, Δ)

3:  $W_{u}\;\leftarrow \;W_{v}$

4:  $W^{\prime }\;\leftarrow \;W_{v}$

5:  *L* ← (*N* + *k*)*v*

6:  n ← 0

7:  **while** n<E **and** $T_{i}<T_{f}$ **do**

8:   **for** *i* ← 0 **to** *i* < *L* **do**

9:     $W_{v}$ ← generateNeighbor$\left(W_{u}\right)$

10:    $A^{\prime }$ ← ConstructSolution

11:    getK $\left(A^{\prime },N,t,k,v\right)$

12:    **for** *i* ← 0 **to** *i* < Δ **do**

13:      $F_{C}\left(G,K\right)$

14:    **end for**

15:    **if** $\tau \left(W_{v}\right)\leq \tau \left(W_{u}\right)$ **then**

16:     $W_{u}\;\leftarrow \;W_{v}$

17:     **if** $\tau \left(W_{u}\right)\leq \tau \left(W^{\prime }\right)$ **then**

18:       $W^{\prime }\;\leftarrow \;W_{u}$

19:     **end if**

20:    **else if** random(0 … 1) $\leq e^{\frac{\tau \left(W_{v}\right)-\tau \left(W_{u}\right)}{T_{i}}}$ **then**

21:      $W_{u}\;\leftarrow \;W_{v}$

22:    **end if**

23:   **end for**

24:    $T_{i}\;\leftarrow \;\alpha \cdot T_{i}$

25:  **end while**

26:   B ← constructMatrix$\left(W^{\prime }\right)$

27:  **return** (B)

28: **end function**

The algorithm MG uses a set of perturbations to the vector $W_{u}$ as its neighborhood function. For this purpose, it chooses two elements *w*_*i*_ and *w*_*j*_, where *w*_*i*_ ≠ *w*_*j*_, and interchanges their values. The new solution formed via this perturbation, which is a neighbor of $W_{u}$, is denoted by $W_{v}$.

Finally, the evaluation function used in GM is *τ*, the number of missing combinations in a created matrix. This function is also used to evaluate the matrices created during the local search.

The time required to execute MG is *O*(*iLF*_*C*_), where $i=\frac{\log_{\alpha }\;T_{f}-\log_{\alpha }\;T_{i}}{\log_{\alpha }\;\alpha }$ is the number of temperature decrements necessary to reach $T_{f}$ and *F*_*C*_ is the time cost of the greedy approach for eliminating columns.

#### Hybrid algorithm ME

The algorithm ME combines the meta-heuristic and exact approaches to solve the OSCAR problem, using a strategy based on the exploration of all possible combinations of Δ columns that can be eliminated from the original matrix. The algorithm proceeds in two phases. First, it chooses a set of Δ columns and removes them from the initial matrix A; the resulting matrix is denoted by $A^{\prime }$ and has dimensions of *N* × (*k* − Δ). Then, the algorithm uses the SA approach to discard *δ* rows from $A^{\prime }$ to construct a possible solution $B^{\prime }$, i.e., a matrix with dimensions of (*N* − *δ*) × (*k* − Δ), for the OSCAR instance at hand. To obtain the best solution B, the algorithm explores all possible combinations of Δ columns and identifies the best matrix B from among all matrices $B^{\prime }$ constructed during the process described above.

Algorithm 11 describes our ME approach. For each possible combination of columns $J_{C}\subset A_{C}$, the algorithm performs a meta-heuristic search to define the set of rows $J_{R}\subset A_{R}$. To determine each element in $J_{C}$, the function GREATERTHANPOLYNOMIAL() [44] is used to systematically generate each different combination of columns.

**Algorithm 11**

1: **function** $ME\left(A,\delta ,\Delta ,T_{i},T_{f},\alpha ,E\right)$

2:  **for** *i* ← 0 **to** i<(kk-Δ) **do**

3:   $J_{C}$ ← GREATERTHANPOLYNOMIAL$\left(J_{C}\right)$

4:   $W_{v}$ ← Initialize(*N*, *δ*)

5:   $W_{u}\;\leftarrow \;W_{v}$

6:   $W^{\prime }\;\leftarrow \;W_{v}$

7:   *L* ← (*N* + *k*)*v*

8:   n ← 0

9:   **while** n<E **and** $T_{i}<T_{f}$ **do**

10:    **for** *j* ← 0 **to** *j* < *L* **do**

11:     $W_{v}$ ← generateNeighbor$\left(W_{u}\right)$

12:     **if** $\tau \left(W_{v}\right)\leq \tau \left(W_{u}\right)$ **then**

13:      $W_{u}\;\leftarrow \;W_{v}$

14:      **if** $\tau \left(W_{u}\right)\leq \tau \left(W^{\prime }\right)$ **then**

15:        $W^{\prime }\;\leftarrow \;W_{u}$

16:      **end if**

17:     **else if** random(0 … 1) $\leq e^{\frac{\tau \left(W_{v}\right)-\tau \left(W_{u}\right)}{T_{i}}}$ **then**

18:      $W_{u}\;\leftarrow \;W_{v}$

19:     **end if**

20:    **end for**

21:    $T_{j}\;\leftarrow \;\alpha \cdot T_{j}$

22:   **end while**

23:   **if** $\tau (W^{{}^{\prime }}<bestS)$ **then**

24:     $bestS\;\leftarrow \;W^{\prime }$

25:     B ← constructMatrix $\left(J_{C},W^{\prime }\right)$

26:   **end if**

27:  **end for**

28:  **return** (B)

29: **end function**

The time required to execute ME is O((kk-Δ)iL), where $i=\frac{\log_{\alpha }\;T_{f}-\log_{\alpha }\;T_{i}}{\log_{\alpha }\;\alpha }$ is the number of temperature decrements necessary to reach $T_{f}$.

#### Hybrid algorithm EM

The algorithm EM also combines the meta-heuristic and exact approaches to solve the OSCAR problem; compared with ME, the difference is that it explores all possible combinations of *δ* rows that can be eliminated from the original matrix. The algorithm proceeds in two phases. First, it chooses a set of *δ* rows and removes them from the initial matrix A; the resulting matrix is denoted by $A^{\prime }$ and has dimensions of (*N* − *δ*) × *k*. Then, the algorithm uses the SA approach to discard Δ columns from $A^{\prime }$ to construct a possible solution $B^{\prime }$, i.e., a matrix with dimensions of (*N* − *δ*) × (*k* − Δ), for the OSCAR instance at hand. To obtain the best solution B, the algorithm explores all possible combinations of *δ* rows and identifies the best matrix B from among all matrices $B^{\prime }$ constructed during the process described above.

Algorithm 12 describes our EM approach. For each possible combination of rows $J_{R}\subset A_{R}$, the algorithm performs a meta-heuristic search to define the set of columns $J_{C}\subset A_{C}$. To determine each element in $J_{R}$, the function GRATERTHANPOLYNOMIAL() [44] is used to systematically generate each different combination of rows.

**Algorithm 12**

1: **function** $EM\left(A,\delta ,\Delta ,T_{i},T_{f},\alpha ,E\right)$

2:  **for** *i* ← 0 **to** i<(kk-δ) **do**

3:   $J_{R}$ ← GREATERTHANPOLYNOMIAL$\left(J_{R}\right)$

4:   $W_{v}$ ← Initialize(*k*, Δ)

5:   $W_{u}\;\leftarrow \;W_{v}$

6:   $W^{\prime }\;\leftarrow \;W_{v}$

7:   *L* ← (*N* + *k*)*v*

8:   *n* ← 0

9:   **while** n<E **and** $T_{i}<T_{f}$ **do**

10:    **for** *i* ← 0 **to** *i* < *L* **do**

11:     $W_{v}$ ← generateNeighbor$\left(W_{u}\right)$

12:     **if** $\tau \left(W_{v}\right)\leq \tau \left(W_{u}\right)$ **then**

13:      $W_{u}\;\leftarrow \;W_{v}$

14:      **if** $\tau \left(W_{u}\right)\leq \tau \left(W^{\prime }\right)$ **then**

15:        $W^{\prime }\;\leftarrow \;W_{u}$

16:      **end if**

17:     **else if** random(0 … 1) $\leq e^{\frac{\tau \left(W_{v}\right)-\tau \left(W_{u}\right)}{T_{i}}}$ **then**

18:       $W_{u}\;\leftarrow \;W_{v}$

19:     **end if**

20:    **end for**

21:    $T_{i}\;\leftarrow \;\alpha \cdot T_{i}$

22:   **end while**

23:   **if** $\tau (W^{{}^{\prime }}<bestS)$ **then**

24:     $bestS\;\leftarrow \;W^{{}^{\prime }}$

25:     B ← constructMatrix$(J_{R},W^{\prime })$

26:   **end if**

27:  **end for**

28:  **return** (B)

29: **end function**

The time required to execute EM is O((NN-Δ)iL), where $i=\frac{\log_{\alpha }\;T_{f}-\log_{\alpha }\;T_{i}}{\log_{\alpha }\;\alpha }$ is the number of temperature decrements necessary to reach $T_{f}$.

In the next section, we demonstrate the performance of our 12 algorithms.

## Experimentation

This section presents the experimental design used to test the performance of the proposed algorithms for solving the OSCAR problem. The methodology consisted of the following steps: 1) A set of benchmark instances was defined. 2) The parameters of the SA algorithm were subjected to a fine-tuning process. 3) The performances of the algorithms were evaluated by using them to solve the benchmark problem instances. 4) A performance comparison against state-of-the-art initialization functions was conducted. 5) The results derived from the algorithms were used to define new upper bounds for existing CAs.

The proposed algorithms were implemented in the C language and compiled using gcc with the optimization option -O3. We used a computer with 72 Intel Xeon 1.6 GHz CPU cores and RAM of 64 GB. The remainder of this section describes the experimental methodology in detail.

### Definition of the benchmarks

This subsection introduces the three benchmarks used to properly test the proposed set of OSCAR algorithms. The benchmark $L_{1}$ (S1 dataset) consists of 12 small CAs, which are described in Table 13, and it is used to analyze the performance of all algorithms presented in this document; then, the algorithms that achieve the best experimental results on this benchmark in terms of both time and solution quality are further tested on the following benchmark. The benchmark $L_{2}$ (S2 dataset), presented in Table 14, consists of 62 CAs; it is an extension of the benchmark presented in [24] such that the adjusted values of *δ* and Δ provide support for the discovery of a greater number of new upper bounds for the related CAs. This benchmark aids in the identification of the OSCAR solver with the best overall experimental performance, and it is also used to compare the results of the proposed OSCAR solvers against other state-of-the art initialization functions. Finally, the benchmark $L_{3}$ (S3 dataset) consists of 820 instances (see Table 15); this benchmark is used to evaluate the quasi-CA construction performance of IPOG-F, a classical and versatile (in the sense that it can rapidly construct any type of CA) greedy algorithm that is widely used in the literature, against the best OSCAR strategies identified in the experiments on the previous benchmarks in terms of both the time required for matrix construction and the quality of the constructed matrices. Table 15 presents the instances included in benchmark $L_{3}$, organized into 20 sets. In each set, one OSCAR instance is defined per value of *k* considered (from 10 to 50), as shown in column 1; the remaining columns show the values for *v*, *t*, *δ*, and Δ, which correspond to the alphabet size, the strength, and the numbers of rows and columns to be eliminated, respectively. We note that the benchmark $L_{3}$ is also characterized by its wide variety of values of the strength *t* and the alphabet size *v*.

**Table 13:** Benchmark $L_{1}$, which is composed of 12 small instances of the OSCAR problem. The column 2 shows the CA uses as initial array, while the columns 3 and 4 show the number of rows *δ* and columns Δ to be shortened, respectively.

Instance	A	*δ*	Δ
1	CA(188;2,140,9)	3	0
2	CA(194;2,36,10)	2	0
3	CA(206;2,78,10)	1	4
4	CA(165;2,14,12)	1	1
5	CA(247;2,18,14)	3	1
6	CA(255;2,18,15)	3	1
7	CA(355;2,12,18)	1	4
8	CA(498;2,29,18)	1	1
9	CA(511;2,22,20)	1	2
10	CA(511;2,22,20)	2	3
11	CA(520;2,22,21)	1	2
12	CA(520;2,22,21)	2	4

**Table 14:** Benchmark $L_{2}$, which is composed of 62 instances of the OSCAR problem. Each instance shows the initial array A, and the number of rows *δ* and columns Δ to be shortened.

Instance	A	*δ*	Δ	Instance	A	*δ*	Δ
1	CA(53;2,52,5)	4	9	32	CA(511;2,22,20)	1	2
2	CA(53;2,52,5)	3	7	33	CA(511;2,22,20)	2	3
3	CA(53;2,52,5)	2	5	34	CA(511;2,22,20)	3	4
4	CA(93;2,113,6)	1	6	35	CA(511;2,22,20)	4	6
5	CA(188;2,140,9)	3	0	36	CA(511;2,22,20)	9	7
6	CA(120;2,80,8)	1	51	37	CA(511;2,22,20)	10	8
7	CA(120;2,80,8)	2	52	38	CA(511;2,22,20)	12	9
8	CA(153;2,99,9)	2	69	39	CA(511;2,22,20)	14	10
9	CA(194;2,36,10)	2	0	40	CA(511;2,22,20)	16	11
10	CA(206;2,78,10)	1	4	41	CA(511;2,22,20)	20	12
11	CA(165;2,14,12)	1	1	42	CA(511;2,22,20)	29	13
12	CA(165;2,14,12)	2	4	43	CA(511;2,22,20)	46	14
13	CA(247;2,18,14)	3	1	44	CA(511;2,22,20)	82	15
14	CA(247;2,18,14)	4	2	45	CA(520;2,22,21)	1	2
15	CA(247;2,18,14)	5	3	46	CA(520;2,22,21)	2	4
16	CA(247;2,18,14)	6	4	47	CA(520;2,22,21)	3	6
17	CA(247;2,18,14)	7	5	48	CA(520;2,22,21)	4	8
18	CA(247;2,18,14)	8	6	49	CA(520;2,22,21)	6	9
19	CA(247;2,18,14)	9	7	50	CA(520;2,22,21)	7	11
20	CA(247;2,18,14)	11	8	51	CA(520;2,22,21)	19	13
21	CA(247;2,18,14)	14	9	52	CA(520;2,22,21)	21	14
22	CA(247;2,18,14)	18	10	53	CA(526;2,24,22)	1	3
23	CA(255;2,18,15)	3	1	54	CA(526;2,24,22)	2	8
24	CA(255;2,18,15)	4	4	55	CA(526;2,24,22)	3	12
25	CA(255;2,18,15)	5	7	56	CA(526;2,24,22)	4	13
26	CA(255;2,18,15)	6	8	57	CA(526;2,24,22)	5	14
27	CA(255;2,18,15)	7	10	58	CA(526;2,24,22)	6	16
28	CA(255;2,18,15)	9	11	59	CA(622;2,26,24)	1	4
29	CA(358;2,20,18)	3	8	60	CA(622;2,26,24)	2	10
30	CA(355;2,12,18)	1	4	61	CA(622;2,26,24)	3	16
31	CA(498;2,29,18)	1	1	62	CA(136;5,68,2)	2	33

**Table 15:** Groups of instances’ sets that form the benchmark $L_{3}$. The column 1 is the identifier of the groups. The columng 2 shows the ranges of *k*, the number of columns. The remaining columns are the alphabet *v*, strength *t*, and rows *δ* and columns Δ to be shortened.

Instance set	A	*v*	*t*	*δ*	Δ
1	10 ≤ *k* ≤ 50	2	2	1	1
2	10 ≤ *k* ≤ 50	2	3	1	1
3	10 ≤ *k* ≤ 50	2	4	1	1
4	10 ≤ *k* ≤ 50	2	5	1	1
5	10 ≤ *k* ≤ 50	3	2	1	1
6	10 ≤ *k* ≤ 50	3	3	1	1
7	10 ≤ *k* ≤ 50	3	4	1	1
8	10 ≤ *k* ≤ 50	4	2	1	1
9	10 ≤ *k* ≤ 50	4	3	1	1
10	10 ≤ *k* ≤ 50	5	2	1	1
11	10 ≤ *k* ≤ 50	5	3	1	1
12	10 ≤ *k* ≤ 50	6	2	1	1
13	10 ≤ *k* ≤ 50	6	3	1	1
14	10 ≤ *k* ≤ 50	2	2	1	0
15	10 ≤ *k* ≤ 50	2	3	1	0
16	10 ≤ *k* ≤ 50	2	4	1	0
17	10 ≤ *k* ≤ 50	2	5	1	0
18	10 ≤ *k* ≤ 50	3	2	1	0
19	10 ≤ *k* ≤ 50	3	3	1	0
20	10 ≤ *k* ≤ 50	3	4	1	0
21	10 ≤ *k* ≤ 50	4	2	1	0
22	10 ≤ *k* ≤ 50	4	3	1	0
23	10 ≤ *k* ≤ 50	5	2	1	0
24	10 ≤ *k* ≤ 50	5	3	1	0
25	10 ≤ *k* ≤ 50	6	2	1	0
26	10 ≤ *k* ≤ 50	6	3	1	0

### Fine-tuning of the parameters of MM

The MM approach is the basis for several of our other approaches. Because this approach uses the SA algorithm, a fine-tuning process is necessary to adjust the values of its parameters to improve its performance. During the tuning process performed in this study, the Markov chain length *L*, the final temperature $T_{f}$, and the initialization function G were fixed; all remaining parameters (i.e., the initial temperature $T_{i}$, the decrement factor *α*, and the maximum number of evaluations E) were subjected to adjustment. Because different neighborhood functions are used in our approach, each with a certain probability of being applied, a fourth parameter was also considered during the tuning process: the application probability of each neighbor function, denoted by P. The goal of this fine-tuning process was to test the performance of MM using different configurations of the parameter values to identify the configuration that yielded the best performance.

The sets of values considered for the parameters $T_{i}$, *α*, and E were {1, 4}, {0.90, 0.99}, and {100*L*, 500*L*}, respectively. In the fine-tuning approach presented in [46, 47], a CA is used as a means of systematically sampling the entire set of parameter value combinations; the method starts at an initial level of interaction *t*, which is used to construct a *CA*(*N*; *t*, *k*, *v*), and *t* is then increased until the generated sample is suitable for the purposes of the experiment. The present study required the smallest possible sample in order to reduce the experimental time; this sample was constructed using an interaction level of *t* = 2. A summary of the final combinations of values tested, derived from the constructed *CA*(4; 2, 3, 2), is shown in Table 16. Meanwhile, the vector of probabilities P used for the initialization functions $S_{i}$ was defined based on solutions to the Diophantine equation *a*_1_*x* + *a*_2_*x* + *a*_3_*x* = 10, following the approach presented in [44]. During this process, each of the 66 solutions to the Diophantine equation was used to generate a possible vector P, in which the probability value for each initialization function *i* was estimated as $\frac{x_{i}}{10}$.

**Table 16:** Different parameter configurations that were tested for the algorithm MM.

Code	$T_{i}$	*α*	E
C1	1	0.90	100*L*
C2	4	0.99	100*L*
C3	4	0.90	500*L*
C4	1	0.99	500*L*

Because we considered 4 different configurations of the values of the parameters $T_{i}$, *α*, and E and 66 configurations of the probability vector P, the experiment to fine-tune MM involved 264 different parameter value configurations. Each configuration was used to solve two instances of the OSCAR problem, specified by (A=CA(31;2,35,4),δ=5,Δ=5) and (A=CA(255;2,18,15),δ=6,Δ=8), with a total of 31 runs per instance, where the value of the solution reported was the best among all the runs.

The results obtained from the fine-tuning process indicated that the optimal parameter values for MM are $T_{i}=4$, *α* = .99, and E=100L and that the desired solution to the Diophantine equation is *a*_1_ = 4, *a*_2_ = 3, and *a*_3_ = 3. This configuration was also used in the algorithms GM, MG, ME, and EM, which also use the meta-heuristic MM approach.

### Evaluation of the 12 proposed algorithms

This section presents the evaluation of the 12 proposed algorithms for solving the OSCAR problem. All algorithms were tested on the smaller set of 12 instances, $L_{1}$, to identify the three best algorithms. Then, the larger set $L_{2}$ was solved using only those three algorithms to further evaluate the general performance of these approaches.

The algorithms were first tested using the benchmark consisting of 12 OSCAR instances derived from 10 CAs taken from the literature. The values *δ* and Δ for these instances were fixed such that the size of the resulting array B would represent a possible new upper bound. In addition, these instances were created such that the size of the search space would permit us to solve them using all 12 algorithms; this was a concern because exact algorithms must explore all possible combinations of rows and columns, and therefore, if the search space is too large, they may require an excessive amount of time.

Table 17 presents the results obtained using each algorithm based solely on the greedy approach (i.e., the algorithms $GG_{CR}$, $GG_{RC}$, and $GG_{{}_{C}^{R}}$) when solving the benchmark $L_{1}$. Note that $GG_{RC}$ and $GG_{{}_{C}^{R}}$ show better performance than $GG_{CR}$; when all instances are considered, the former algorithms result in equal or fewer missing *t*-wise combinations compared with the latter. Therefore, the findings show that it is beneficial to eliminate rows before columns (as in $GG_{RC}$ and $GG_{{}_{C}^{R}}$) when working with initial matrices that are already CAs. This is because when rows are removed from the matrix, those that contribute the least to the CA are chosen for deletion, and the *t*-wise combinations that are lost as a result can subsequently be compensated for by eliminating the columns that produce them. Meanwhile, although the time performance of $GG_{{}_{C}^{R}}$ is superior to that of the others for this particular set of instances, it will worsen rapidly with increasing values of *δ* and Δ. In general, the time performance of $GG_{{}_{C}^{R}}$ will be the worst among the three greedy approaches, as indicated by the theoretical complexities presented alongside the definitions of these algorithms, mainly because of the greater number of calls to the greedy strategies for eliminating rows and columns.

**Table 17:** Results obtained when solving $L_{1}$ using the algorithms $GG_{CR}$, $GG_{RC}$, and $GG_{{}_{C}^{R}}$.

Quality of solution τ(B)				Time (sec.)			
Instance	$GG_{{}_{C}^{R}}$	GE	EG	Instance	$GG_{{}_{C}^{R}}$	GE	EG
1	110	110	110	1	0.5	0.5	0.5
2	2	2	2	2	0.1	0.1	0.1
3	85	74	74	3	0.2	1.2	0.3
4	43	43	43	4	0.1	0.1	0.1
5	193	187	191	5	0.2	0.3	0.1
6	268	267	267	6	0.1	0.3	0.3
7	12	9	9	7	0.1	0.2	0.1
8	95	92	92	8	0.2	0.5	0.2
9	87	82	82	9	0.2	0.3	0.2
10	153	143	142	10	0.2	0.5	0.2
11	109	108	108	11	0.2	0.3	0.2
12	168	161	161	12	0.2	0.5	0.3

Table 18 shows the results obtained using the hybrid approaches that combine the greedy strategy with either the exact approach or the meta-heuristic approach (i.e., the algorithms GE, EG, and GM) when solving the benchmark $L_{1}$. An increase in running time is observed for these approaches, mainly due to the use of the more elaborate strategies of the exact and meta-heuristic algorithms. However, the results achieved also improve upon some of the results obtained by the solely greedy algorithms. All of these hybrid algorithms achieve the same results; however, the average time increase for GM is much greater than that for the algorithms that include exact strategies. Let’s point out that the small amounts of times appearing in the exact approach are indeed a result from its expected theoretical behavior.

**Table 18:** Results obtained when solving $L_{1}$ using the algorithms GE, EG, and GM.

Quality of solution τ(B)				Time (sec.)			
Instance	$GG_{{}_{C}^{R}}$	GE	EG	Instance	$GG_{{}_{C}^{R}}$	GE	EG
1	110	110	110	1	0.5	41301.2	2507.3
2	2	2	2	2	0.2	0.2	0.2
3	74	74	74	3	97742.5	2.7	273.2
4	43	43	43	4	0.1	0.1	9225.3
5	187	187	187	5	0.2	1731.2	9286.5
6	267	267	267	6	0.2	2035.7	12507.5
7	9	9	9	7	2.3	0.2	23.5
8	105	105	105	8	2.5	2.7	927.5
9	82	82	82	9	10.8	1.2	867.5
10	142	142	142	10	78.5	268.2	608.2
11	108	108	108	11	15.3	1.5	1123.5
12	161	161	161	12	502.8	280.3	837.3

Table 19 shows the results for another set of hybrid approaches, all involving the meta-heuristic strategy in combination with either the exact approach or the greedy approach (i.e., the algorithms MG, ME, and EM), when solving the benchmark $L_{1}$. From these results and the previous ones shown in Table 18 for GM, it can be seen that the algorithms GM, MG, ME, and EM all find solutions with a comparable number of missing *t*-wise combinations to those in the solutions created by the other (exact or greedy) approaches. However, it should be noted that the initial matrices used in these algorithms were the best solutions obtained by a greedy algorithm, and in most cases, the differences in the number of missing *t*-wise combinations between these initial matrices and the results reported by the hybrid algorithms are nearly zero. These findings indicate that the contribution of these hybrid approaches is minimal.

**Table 19:** Results obtained when solving $L_{1}$ using the algorithms MG, ME, and EM.

Quality of solution τ(B)				Time (sec.)			
Instance	$GG_{{}_{C}^{R}}$	GE	EG	Instance	$GG_{{}_{C}^{R}}$	GE	EG
1	110	110	110	1	5051.3	3252.3	15357.2
2	2	2	2	2	11300.8	2352.5	7542.5
3	94	94	94	3	47.2	5472.3	15.2
4	63	63	43	4	1399.7	0.2	11615.2
5	225	187	187	5	9850.2	9242.7	12503.2
6	308	267	267	6	12778.2	2582.3	15253.2
7	21	21	21	7	2.7	5.2	47.5
8	105	105	105	8	5082.5	1.2	5.3
9	93	82	82	9	35.2	15.2	82.5
10	181	181	181	10	35.7	17.2	42.7
11	108	108	108	11	12323.7	1.7	15.2
12	184	184	184	12	32.5	48.3	82.5

Table 20 shows the results of solving the benchmark $L_{1}$ using the algorithms MM, $EE_{CR}$, and $EE_{RC}$. Note that $EE_{CR}$ exhibits better performance than $EE_{RC}$ because there are more possible ways to select rows than columns. However, exhaustive search algorithms are impractical for finding a solution to an OSCAR instance except when the values of *δ* and Δ are both quite small. The exact algorithm $EE_{CR}$ is more suitable when *N* − *δ* > *k* − Δ since a greater portion of the search space is defined by *N* − *δ*; otherwise, $EE_{RC}$ behaves better. However, the execution time of the proposed exact algorithms grows with the desired degree of reduction for a given instance, and they can become infeasible.

**Table 20:** Results obtained when solving $L_{1}$ using the algorithms MM, $EE_{CR}$, and $EE_{RC}$.

Quality of solution τ(B)				Time (sec.)			
Instance	$GG_{{}_{C}^{R}}$	GE	EG	Instance	$GG_{{}_{C}^{R}}$	GE	EG
1	110	110	110	1	0.3	32.8	3.7
2	2	2	2	2	0.3	0.2	0.2
3	74	74	74	3	0.3	12142.7	1252.3
4	43	43	43	4	0.5	0.1	1.2
5	187	187	187	5	1.5	1732.3	15242.2
6	267	267	267	6	5.7	1050.5	12345.2
7	9	9	9	7	0.3	135.2	15.5
8	105	105	105	8	0.3	8.5	82.3
9	82	82	82	9	0.3	0.5	5.2
10	142	142	142	10	0.3	23247.3	207872.5
11	108	108	108	11	1.2	7.2	63.5
12	161	161	161	12	323.2	232829.8	697482.5

Finally, some additional important observations are noted in the following. First, for every instance, the solutions obtained by the algorithms GE and EG have the same number of missing *t*-wise combinations. When *N* − *δ* > *k* − Δ, EG requires more time than GE; similarly, when *N* − *δ* < *k* − Δ, GE requires more time than EG. These findings suggest that GE is appropriate when *N* − *δ* > *k* − Δ and that EG is appropriate when *N* − *δ* < *k* − Δ.

Second, as *δ* and Δ increase for a given array A, the number of missing *t*-wise combinations produced by the pure greedy algorithms increases in comparison with the results of the hybrid algorithms that include exact strategies, i.e., EG and GE. For example, for the instances with the input array *CA*(255; 2, 18, 15), we note that the solution obtained by $GG_{{}_{C}^{R}}$ when *δ* = 3 and Δ = 1 has 267 missing *t*-wise combinations, whereas the solution obtained by GE has 257 missing *t*-wise combinations; similarly, when *δ* = 9 and Δ = 11, the solution obtained by $GG_{{}_{C}^{R}}$ has 65 missing *t*-wise combinations, whereas the solution obtained by GE has 61 missing *t*-wise combinations. It can be inferred that the inclusion of an exact strategy contributes to reducing the number of missing *t*-wise combinations, at the cost of an increase in the time required to build the matrix.

Third, and most importantly, the algorithms that showed the best performance in the experiment were $GG_{{}_{C}^{R}}$, GE and EG; all of them obtained comparable solutions, with only small differences in both quality and time cost. The algorithms that include meta-heuristic strategies consumed considerably more time but showed little difference in the quality of their solutions, whereas the exact approaches are too expensive for large values of *δ* and Δ.

To further evaluate the proposed approaches, the algorithms $GG_{{}_{C}^{R}}$, GE and EG were used to solve the benchmark $L_{2}$, which includes larger CAs. Table 21 summarizes the results obtained when solving $L_{2}$. In addition to this experiment, an instance specified by the array A=CA(136;5,68,2) and values of *δ* = 2 and Δ = 33 was also solved using the meta-heuristic algorithm MM; this algorithm produced a solution B with zero missing *t*-wise combinations, meaning that the approach constructed a new CA of the form *CA*(134; 5, 35, 2). This last result serves as evidence that an approach based on seeking a solution to the OSCAR problem can also be used to construct CAs.

**Table 21:** Results obtained when solving $L_{2}$ using the algorithms $GG_{{}_{C}^{R}}$, GE and EG.

Quality of solution τ(B)				Time (sec.)			
Instance	$GG_{{}_{C}^{R}}$	GE	EG	Instance	$GG_{{}_{C}^{R}}$	GE	EG
1	272	-	267	1	0.1	-	349.7
2	229	-	216	2	0.1	-	32.5
3	173	172	172	3	0.1	1070.3	1.9
4	154	-	154	4	0.2	-	1.2
6	33	-	33	6	0.2	-	0.7
7	60	-	60	7	0.2	-	28.9
8	60	-	60	8	0.3	-	7357.2
12	41	40	40	12	0.2	1.5	3.5
14	214	214	214	14	0.3	0.2	100350.2
15	227	227	-	15	0.2	5.2	-
16	228	226	-	16	0.2	14.2	-
17	219	215	-	17	0.2	37.2	-
18	199	199	-	18	0.1	65.2	-
19	180	175	-	19	0.1	93.5	-
20	177	168	-	20	0.1	108.3	-
21	168	162	-	21	0.2	100.7	-
22	163	155	-	22	0.1	74.7	-
24	216	215	-	24	0.1	15.7	-
25	142	139	-	25	0.2	102.3	-
26	130	127	-	26	0.1	116.7	-
27	79	74	-	27	0.1	77.5	-
28	65	61	-	28	0.1	45.2	-
29	99	98	98	29	0.1	1088.2	7158.7
34	188	186	-	34	0.3	367.8	-
35	182	174	-	35	0.5	2433.5	-
36	370	363	-	36	0.5	5299.7	-
37	343	336	-	37	0.5	8503.7	-
38	346	337	-	38	0.5	15335.2	-
39	335	320	-	39	0.8	15820.8	-
40	309	292	-	40	0.8	13832.8	-
41	301	286	-	41	0.8	9502.3	-
42	346	339	-	42	0.8	8112.7	-
43	455	434	-	43	1.2	3703.5	-
44	681	641	-	44	1.2	2144.2	-
47	180	173	173	47	0.2	4003.2	45270.3
48	161	161	-	48	0.5	11791.7	-
49	208	201	-	49	0.7	16457.2	-
50	154	140	-	50	0.7	15723.8	-
51	275	263	-	51	1.2	8457.2	-
52	235	212	-	52	1.5	4220.5	-
53	140	140	140	53	0.3	242.7	1.7
54	140	140	140	54	0.5	50557.5	306.7
55	102	98	-	55	0.5	90420.2	-
56	110	106	-	56	0.7	60493.2	-
57	110	103	-	57	0.8	51802.5	-
58	74	67	-	58	0.8	10737.5	-
59	74	74	74	59	0.5	3045.5	2.8
60	100	67	67	60	0.8	1073936.3	623.3
61	42	42	-	61	1.2	300241.7	-
62	832	-	353	62	352.5	-	12552.3

### Performance comparison with state-of-the-art initialization algorithms

This subsection evaluates the performance of the proposed OSCAR approaches against the performance of several state-of-the-art initialization functions. For this purpose, the initialization functions described in the related work section are considered, and their results are compared with the best solutions obtained using the approaches proposed in this work.

The performance comparison was performed as follows. The benchmark $L_{2}$ was chosen as the set of instances to be used in this evaluation. First, the OSCAR algorithms proposed in this work were used to solve the benchmark, and the best matrix B among all of the results was obtained for each instance. Then, the initialization functions, denoted by $I_{i}$, were used to construct arrays $S_{i}$ of the same dimensions as the matrices derived by solving the OSCAR instances; i.e., for each instance, we constructed an array S with *N* − *δ* rows and *k* − Δ columns. Once all of the solutions generated by the OSCAR algorithms and the state-of-the-art initialization functions had been obtained, they were evaluated with regard to the function *τ*, i.e., the number of missing *t*-wise combinations in each newly constructed matrix. Table 22 summarizes the results of this experiment. Column one shows the identifier of each instance in $L_{2}$, column two shows the number of missing *t*-wise combinations in the best solution obtained using the OSCAR approaches, and columns three to six present the numbers of missing *t*-wise combinations in the solutions derived using the state-of-the-art initialization functions $I_{i}$. Note that for the last problem instance, one of the proposed OSCAR approaches (the meta-heuristic algorithm MM) was able to construct a CA, as seen from the fact that the new matrix has zero missing *t*-wise combinations.

**Table 22:** Quality of solutions measured as missing *t*-wise combinations τ, and obtained by the best solution B from the proposed approaches, and the initialization functions.

Instance	τ(B)	$\tau (S_{1})$	$\tau (S_{2})$	$\tau (S_{3})$	$\tau (S_{4})$
1	267	2892	2188	2834	8820
2	216	2971	2168	2979	9680
3	173	3035	2263	3148	10580
4	154	15106	10357	14731	84270
5	110	77860	62692	78120	347760
6	33	3787	3503	3783	10976
7	60	3504	3251	3553	10192
8	60	5110	4798	5140	15120
9	2	8876	8132	8854	27540
10	74	33573	29110	33682	119880
11	43	3511	3950	3455	4752
12	40	2010	2371	2017	2640
13	187	7501	8252	7410	11648
14	214	6648	7488	6617	10192
15	227	5841	6692	5855	8918
16	226	5046	5797	5071	7644
17	215	4380	5103	4403	6552
18	199	3703	4456	3718	5460
19	175	3109	3843	3087	4550
20	168	2561	3151	2542	3640
21	162	1200	1649	1179	1638
22	155	869	1268	877	1092
23	215	9850	11201	9781	13440
24	139	6541	7807	6552	8820
25	267	3971	4773	3937	5250
26	127	3257	4147	3260	4200
27	74	2021	2601	2033	2520
28	61	1503	2049	1523	1890
29	98	7032	8821	7065	9180
30	9	2979	3957	2941	3672
31	105	25933	29051	25998	55692
32	82	21042	25047	20854	34200
33	142	18960	22757	18893	30780
34	186	16987	20643	16837	27360
35	174	13295	16773	13275	21280
36	363	11720	14674	11791	18620
37	336	10240	13145	10212	15960
38	337	8784	11640	8845	13680
39	320	7435	10103	7475	11400
40	292	6251	8571	6261	9500
41	286	5173	7263	5149	7600
42	339	4233	6102	4207	6080
43	434	3392	5079	3415	4560
44	641	2771	4145	2802	3420
45	108	25471	30741	25577	37800
46	161	20643	25142	20625	30204
47	173	16203	20203	16201	23520
48	161	12253	15671	12304	17640
49	201	10595	13745	10597	15120
50	140	7450	10182	7432	10500
51	263	4971	7102	5003	6720
52	212	3871	5735	3900	5040
53	140	33977	40780	33997	46200
54	140	19485	23693	19448	25872
55	98	10715	13837	10675	13860
56	106	8840	12033	8937	11550
57	103	7292	9982	7308	9240
58	67	4537	6505	4541	5544
59	74	44732	54182	44972	60720
60	67	23348	29560	23340	30912
61	42	8678	12371	8723	11040
**62**	**0**	**108240**	**112362**	**113457**	**6314350**

### Performance comparison with IPOG-F, a state-of-the-art CA construction approach

The experiment presented here involves the comparison of the $GG_{RC}$ and EG strategies against the state-of-the-art IPOG-F algorithm for CA construction. The goal in this experiment was to evaluate the performance when constructing CAs and/or quasi-CAs using IPOG-F, a fast greedy algorithm for CA construction that is widely used in the literature and is versatile in the sense that it can rapidly construct any type of CA. The $GG_{RC}$ and EG strategies are among the best of the proposed OSCAR solvers, as indicated by the experiments on the previous benchmarks. Both of these strategies were compared against IPOG-F in terms of the matrix construction time and the matrix quality (i.e., the number of missing *t*-combinations). Table 23 summarizes the results of this comparison on $L_{3}$. Column 1 lists each set of instances in the benchmark. Columns 2 to 4 present the accumulated solution quality (i.e., the accumulated number of missing *t*-wise combinations) per set for each strategy. Columns 5 to 7 report the accumulated time per set and strategy.

**Table 23:** Summary of the results of evaluating the performance of algorithms *E*_1_ = IPOG-F, $E_{2}=GG_{RC}$, and $E_{3}=EG$_3_, over the benchmark $L_{3}$. The performance is measured in the missing *t*-wise combinations, and in the time (in seconds) spent to find it.

Instance set	Missings			Time		
	*E*_1_	*E*_2_	*E*_3_	*E*_1_	*E*_2_	*E*_3_
1	1993	237	236	17,18	0,27	0,28
2	16000	27	23	60,46	1,30	8,29
3	87315	6	5	177,25	19,41	446,69
4	228909	1	0	8900,10	450,54	25384,61
5	9501	62	62	38,97	0,28	0,54
6	69913	8	5	219,88	3,22	64,30
7	247416	0	0	2758,92	149,86	21925,19
8	21344	20	20	45,45	0,43	1,24
9	175871	1	0	76,60	7,00	350,69
10	35872	30	30	23,94	0,41	1,90
11	291423	0	0	104,53	18,63	1407,30
12	51142	18	18	32,10	0,62	3,83
13	506926	0	0	176,10	41,27	4483,16
14	325	286	286	17,18	0,13	0,23
15	118	87	87	60,46	1,15	9,88
16	66	58	58	177,25	20,08	469,28
17	61	45	45	8900,10	456,45	32090,95
18	112	108	108	38,97	0,19	0,54
19	69	59	59	219,88	3,32	70,20
20	52	40	40	2758,92	149,98	23156,30
21	84	82	82	45,45	0,29	1,23
22	53	46	46	76,60	7,53	363,88
23	96	90	90	23,94	0,37	3,30
24	47	43	43	104,53	19,80	1488,70
25	63	63	63	32,10	0,54	4,60
26	48	43	43	176,10	42,38	4531,78

The experiment reported in this section was conducted to test IPOG-F as an approach for constructing CAs and/or quasi-CAs. The results shown in Table 23 reveal that the matrices constructed using $EG_{RC}$ have up to 90% fewer missing *t*-wise combinations than those constructed using IPOG-F. In addition, $EG_{RC}$ could generate CAs in 40 of the 820 OSCAR instances by reducing the number of missing *t*-wise combinations to 0, whereas IPOG-F failed to obtained any CA with the desired numbers of rows and columns. Finally, $EG_{RC}$ achieved better running times than IPOG-F for small values of *v* and *t*; however, the time performance of $EG_{RC}$ rapidly worsened with increasing values of the alphabet size and strength. By contrast, the $GG_{RC}$ strategy achieved time consumption results similar to those of IPOG-F while also improving the solution quality, making it a better choice than IPOG-F for the construction of quasi-CAs.

### Applications of the proposed approaches for solving the OSCAR problem

In this subsection, we demonstrate that the matrices constructed by solving the OSCAR problem can be used as initial matrices for meta-heuristics for CA construction to assist in the construction of better matrices. For this purpose, the outputs of the initialization functions described in the related work section and the best solutions obtained using the proposed OSCAR approaches were used as the initial matrices for a meta-heuristic reported in [22].

Table 24 shows the new upper bounds for *CAN*(*t*, *k*, *v*) obtained using our proposed methodology. The second column shows the new CA bounds obtained when using the best matrices generated by the proposed OSCAR algorithms as the initial matrices for the meta-heuristic algorithm, and the third column shows the previous upper bounds for those CAs. The best produced solution for each specific instance of the OSCAR problem was used as the initial matrix for the meta-heuristic CA construction algorithm reported in [22], which is also based on the SA algorithm. Because of the small number of missing *t*-wise combinations in all of the produced initial matrices, the performance of the meta-heuristic algorithm was improved. The results define new upper bounds on *CAN*(*t*, *k*, *v*) for several CAs.

**Table 24:** New upper bounds.

CAN	New	Previous	CAN	New	Previous
*CAN*(2, 43, 5)	49	50	*CAN*(355; 2, 12, 18)	355	358
*CAN*(2, 45, 5)	50	52	*CAN*(354; 2, 8, 18)	354	355
*CAN*(2, 47, 5)	51	53	*CAN*(2, 28, 18)	497	498
*CAN*(2, 29, 8)	119	120	*CAN*(2, 20, 20)	510	511
*CAN*(2, 28, 8)	118	120	*CAN*(2, 19, 20)	509	511
*CAN*(2, 25, 8)	114	120	*CAN*(2, 18, 20)	508	511
*CAN*(2, 24, 8)	113	120	*CAN*(2, 16, 20)	507	511
*CAN*(2, 21, 8)	112	120	*CAN*(2, 15, 20)	502	511
*CAN*(2, 25, 9)	144	153	*CAN*(2, 14, 20)	501	511
*CAN*(2, 26, 9)	145	153	*CAN*(2, 13, 20)	499	511
*CAN*(2, 30, 9)	151	153	*CAN*(2, 12, 20)	497	511
*CAN*(2, 140, 9)	185	188	*CAN*(2, 11, 20)	495	511
*CAN*(2, 107, 6)	92	92	*CAN*(2, 10, 20)	491	511
*CAN*(2, 36, 10)	192	194	*CAN*(2, 9, 20)	482	511
*CAN*(2, 74, 10)	205	206	*CAN*(2, 8, 20)	465	511
*CAN*(2, 13, 12)	164	165	*CAN*(2, 7, 20)	429	511
*CAN*(2, 10, 12)	163	165	*CAN*(2, 20, 21)	519	520
*CAN*(2, 17, 14)	244	247	*CAN*(2, 18, 21)	518	520
*CAN*(2, 16, 14)	243	247	*CAN*(2, 16, 21)	517	520
*CAN*(2, 15, 14)	242	247	*CAN*(2, 14, 21)	516	520
*CAN*(2, 14, 14)	241	247	*CAN*(2, 13, 21)	514	520
*CAN*(2, 13, 14)	240	247	*CAN*(2, 11, 21)	513	520
*CAN*(2, 12, 14)	239	247	*CAN*(2, 9, 21)	501	520
*CAN*(2, 11, 14)	238	247	*CAN*(2, 8, 21)	499	520
*CAN*(2, 10, 14)	246	247	*CAN*(2, 21, 22)	525	526
*CAN*(2, 9, 14)	233	247	*CAN*(2, 16, 22)	524	526
*CAN*(2, 8, 14)	229	247	*CAN*(2, 12, 22)	523	526
*CAN*(2, 17, 15)	252	255	*CAN*(2, 11, 22)	522	526
*CAN*(2, 14, 15)	251	255	*CAN*(2, 10, 22)	521	526
*CAN*(2, 11, 15)	250	255	*CAN*(2, 8, 22)	520	526
*CAN*(2, 10, 15)	249	255	*CAN*(2, 22, 24)	621	622
*CAN*(2, 8, 15)	248	255	*CAN*(2, 16, 24)	620	622
*CAN*(2, 7, 15)	246	255	*CAN*(2, 10, 24)	619	622

## Conclusions

The present work has indirect implications for the interaction testing of software by aiding in the construction of tests of economical size (a feasible number of test cases). In particular, this paper presents and analyzes strategies for the construction of arrays with sufficiently few missing combinations to be considered quasi-CAs. Such arrays are constructed by solving the problem known as the Optimal Shortening of Covering ARrays (OSCAR) problem. The development of these strategies is motivated by the fact that the arrays thus produced can be used as excellent initialization matrices for algebraic or meta-heuristic approaches for the construction of CAs, which are mathematical objects that have broad applications in the testing of software components.

This work presents an analysis of twelve different strategies for solving the OSCAR problem. Five of them correspond to greedy and exact approaches previously described in the literature, whereas the remaining seven algorithms are newly proposed here. The new approaches involve the use of simulated annealing and hybridization in their design. We note that this work also provides pseudocodes for the design of all presented algorithms, including, for the first time, the designs for the greedy approaches, which have been only briefly described in previous works. In addition, to test these strategies, three new OSCAR benchmarks with more than 1,000 instances have been designed, representing a considerable improvement over the previously reported 20-instance benchmark in terms of both size and variety in the values of the strength and alphabet size parameters, *t* and *v*, respectively.

The experimental design developed for the comparative analysis involved all three proposed benchmarks. The first benchmark, which consists of small instances, was solved using all twelve strategies: three greedy algorithms $\left\{GG_{CR},GG_{RC},GG_{{}_{C}^{R}}\right\}$, two exact algorithms $\left\{EE_{CR},EE_{RC}\right\}$, one meta-heuristic algorithm {MM}, and six hybrid approaches {GE,EG,GM,MG,ME,EM}. Using this benchmark, the algorithms were compared in terms of running time and solution quality (measured as the number of missing *t*-wise combinations in each constructed array). As expected, the results showed that the greedy algorithms were the fastest, the exact algorithms yielded the best solutions, and the meta-heuristic provided a balance between quality and time. It was also observed that the solution quality of the pure greedy algorithms worsened with increasing instance size, but this situation could be addressed through the use of hybrid algorithms. The hybrid algorithms involving a mixture of greedy and exact approaches had higher running times but also higher solution quality. The first experiment indicated that hybrid algorithms involving a mixture of the meta-heuristic and greedy strategies are a viable alternative. Such strategies had somewhat higher running times for array construction but resulted in fewer missing *t*-wise combinations than the hybrid greedy approaches, mainly when the numbers of rows and columns to be deleted were high. In terms of solution quality, the experimental results indicated that the best algorithms were GE and EG because they yielded solutions with as few missing *t*-wise combinations as the exact approaches $EE_{RC}$ and $EE_{CR}$ but in less time. In terms of running time, the experiment indicated that the best algorithms were $GG_{{}_{C}^{R}}$ and $GG_{RC}$ because they were faster than any other algorithms while maintaining an acceptable solution quality; however, we note that for larger instances, the time performance of $GG_{{}_{C}^{R}}$ will be worse than that of $GG_{RC}$ because it is more strongly affected by the instance size and the numbers of rows and columns to be removed.

The second benchmark was used to perform an in-depth analysis of some of the best strategies, namely, $GG_{{}_{C}^{R}}$, GE and EG. This experiment tested the performance of these algorithms on larger OSCAR instances to yield a better understanding of their behavior. The best solutions were still produced by the hybrid greedy-exact approaches GE and EG, but in the latter approach the time increased exponentially. By contrast, the pure greedy algorithm $GG_{{}_{C}^{R}}$ continued to be fast, and its solutions only slightly deviated from those of the hybrid algorithms. After this analysis, the same benchmark was used to compared the best results from these approaches against the initialization functions generated using state-of-the-art methods. The experimental results showed that in all instances, the number of missing *t*-wise combinations was reduced by approximately 90% in the matrices constructed using the proposed approach in comparison with those taken from the literature.

Finally, an experiment was conducted using the third benchmark to test IPOG-F as an approach for constructing CAs and/or quasi-CAs. The results revealed that with $EG_{RC}$, the number of missing *t*-wise combinations was reduced by up to 90% compared with IPOG-F. Moreover, it was found that $EG_{RC}$ could obtain CAs in 40 of the 820 OSCAR instances by reducing the number of missing *t*-wise combinations to 0, whereas IPOG-F failed to obtain any CA with the desired numbers of rows and columns. Finally, it was observed that the running time of $EG_{RC}$ was better than that of IPOG-F for small values of *v* and *t* but worsened rapidly with increasing values of the alphabet size and strength. By contrast, the $GG_{RC}$ strategy achieved running times similar to those of IPOG-F while also improving the solution quality, making it a better choice than IPOG-F for the construction of quasi-CAs.

A major drawback of some of the proposed approaches (with the exception of the greedy ones) is the time consumed to solve the problem, which increases with the numbers of rows and columns to be eliminated. Moreover, the experimental design could be improved to test a wider range of possible values to adjust the meta-heuristic and investigate a wider number of strategies. The ranges of values of the alphabet size and strength parameters should be extended to further probe the resulting changes in performance of the different strategies. Future work should also address the lack of an in-depth analysis of the use of the meta-heuristic approach to properly characterize its region of importance. In general, a more extensive characterization study could provide better insight into the behavior of these strategies, and this remains as future work.

## Supporting information

S1 DatasetBenchmark $L_{1}$.(ZIP)

S2 DatasetBenchmark $L_{2}$.(ZIP)

S3 DatasetBenchmark $L_{3}$.(ZIP)
